# Combining genetic markers, on-farm information and infrared data for the in-line prediction of blood biomarkers of metabolic disorders in Holstein cattle

**DOI:** 10.1186/s40104-024-01042-3

**Published:** 2024-06-09

**Authors:** Lucio F. M. Mota, Diana Giannuzzi, Sara Pegolo, Hugo Toledo-Alvarado, Stefano Schiavon, Luigi Gallo, Erminio Trevisi, Alon Arazi, Gil Katz, Guilherme J. M. Rosa, Alessio Cecchinato

**Affiliations:** 1 Department of Agronomy, Food, Natural resources, Animals and Environment (DAFNAE), University of Padova, Legnaro, Padova 35020 Italy; 2 Department of Genetics and Biostatistics, School of Veterinary Medicine and Zootechnics, National Autonomous University of Mexico, Ciudad Universitaria, Mexico City, 04510 Mexico; 3 Department of Animal Science, Food and Nutrition (DIANA) and the Romeo and Enrica Invernizzi Research Center for Sustainable Dairy Production (CREI), Faculty of Agricultural, Food, and Environmental Sciences, Università Cattolica del Sacro Cuore, Piacenza, 29122 Italy; 4 Afimilk LTD, Afikim, 15148 Israel; 5 Department of Animal and Dairy Sciences, University of Wisconsin, Madison, WI 53706 USA

**Keywords:** Blood metabolites, Dairy cattle, Data integration, Feature selection, Metabolic disorders, NIR, Precision livestock farming

## Abstract

**Background:**

Various blood metabolites are known to be useful indicators of health status in dairy cattle, but their routine assessment is time-consuming, expensive, and stressful for the cows at the herd level. Thus, we evaluated the effectiveness of combining in-line near infrared (NIR) milk spectra with on-farm (days in milk [DIM] and parity) and genetic markers for predicting blood metabolites in Holstein cattle. Data were obtained from 388 Holstein cows from a farm with an AfiLab system. NIR spectra, on-farm information, and single nucleotide polymorphisms (SNP) markers were blended to develop calibration equations for blood metabolites using the elastic net (ENet) approach, considering 3 models: (1) Model 1 (M1) including only NIR information, (2) Model 2 (M2) with both NIR and on-farm information, and (3) Model 3 (M3) combining NIR, on-farm and genomic information. Dimension reduction was considered for M3 by preselecting SNP markers from genome-wide association study (GWAS) results.

**Results:**

Results indicate that M2 improved the predictive ability by an average of 19% for energy-related metabolites (glucose, cholesterol, NEFA, BHB, urea, and creatinine), 20% for liver function/hepatic damage, 7% for inflammation/innate immunity, 24% for oxidative stress metabolites, and 23% for minerals compared to M1. Meanwhile, M3 further enhanced the predictive ability by 34% for energy-related metabolites, 32% for liver function/hepatic damage, 22% for inflammation/innate immunity, 42.1% for oxidative stress metabolites, and 41% for minerals, compared to M1. We found improved predictive ability of M3 using selected SNP markers from GWAS results using a threshold of > 2.0 by 5% for energy-related metabolites, 9% for liver function/hepatic damage, 8% for inflammation/innate immunity, 22% for oxidative stress metabolites, and 9% for minerals. Slight reductions were observed for phosphorus (2%), ferric-reducing antioxidant power (1%), and glucose (3%). Furthermore, it was found that prediction accuracies are influenced by using more restrictive thresholds (−log_10_(*P*-value) > 2.5 and 3.0), with a lower increase in the predictive ability.

**Conclusion:**

Our results highlighted the potential of combining several sources of information, such as genetic markers, on-farm information, and in-line NIR infrared data improves the predictive ability of blood metabolites in dairy cattle, representing an effective strategy for large-scale in-line health monitoring in commercial herds.

**Supplementary Information:**

The online version contains supplementary material available at 10.1186/s40104-024-01042-3.

## Background

The high energy requirements of milk production can lead to metabolic disorders in dairy cows during early lactation and throughout the lactation period [1]. Experiencing stress can trigger lipolysis and proteolysis to support high milk yields [2, 3]. As a result, metabolic stress leads to an increment in blood levels of haptoglobin, non-esterified fatty acids (NEFA), β-hydroxybutyrate (BHB), ceruloplasmin (CuCp), and globulins, followed by a reduction in glucose, paraoxonase (PON) and albumin levels [1]. Variations of blood minerals are also observed, which can be used as biological markers, specifically calcium as a primary indicator of milk fever, and sodium, potassium, and zinc as markers of systemic inflammation and oxidation. These alterations in blood metabolite levels are directly linked with the main metabolic disorders (energy imbalances, ketosis, and milk fever) and negatively impact dairy herd profitability and production by affecting milk production, reproductive efficiency, and overall herd health [4, 5]. Consequently, there has been a growing interest in addressing these metabolic issues during the lactation phase to improve the health and resilience of dairy cows.

Traditionally, the metabolic evaluation of a herd is monitored mainly through blood metabolic profiling [6], allowing the identification and selection of resilient cows less predisposed to developing metabolic disorders. However, its assessment at either an individual or herd level is expensive and time-consuming. Despite this, there has been increased attention on metabolic stress in lactating dairy cows due to its harmful effects on the profitability and sustainability of dairy herds [7]. In this context, noninvasive high-throughput phenotyping technologies based on milk infrared spectroscopy have been applied to predict variation in blood metabolites [8–11]. This is mainly possible because the detailed composition of raw milk reflects the health and nutritional status of dairy cows, and the disruption of metabolic homeostasis is reflected in alterations to these components [12, 13]. Milk spectral analysis is a promising method for assessing the metabolic status of dairy cows on a large scale due to the interaction between metabolic status and milk compounds, mainly fat and protein [14]. In this regard, automated milk quality sensors are used to check collected milk’s quality and look for any health biomarkers in real-time at the herd level [10, 15]. These in-line near-infrared (NIR) milk sensors play a vital role in herd management technologies, especially in monitoring cows’ nutrition and detecting metabolic alterations [16] by examining changes in milk composition over time. Giannuzzi et al. [10], with a first attempt using various machine learning methods, explored the possibility of predicting blood metabolic profile from the milk of individual cows using an in-line NIR spectroscopy milk analyzer, obtaining low to moderate predictions (from 0.30 to 0.65).

Considering the complex nature of metabolic stress during the lactation period, it is worthwhile to consider multiple sources of information. Integrating different layers of information has already been proposed to enhance the development of more accurate predictive models, increasing the capability to detect metabolic disturbances in dairy herds [8]. Early and accurate detection of cows prone to metabolic disorders is crucial to building strategies to support farm management and breeding decisions to detect metabolic disorders efficiently. Therefore, this study was conducted to assess the potential benefits of integrating in-line NIR milk sensor infrared information with on-farm data (DIM and parity) and genetic markers for predicting the blood metabolic profile in Holstein cattle.

## Materials and methods

### Field data

A total of 388 Holstein cows from a single herd in northern Italy (Piacenza province) were sampled for blood. These cows received a twice-daily feeding regimen consisting of a diet primarily composed of corn silage and sorghum. Energy-protein supplementation was provided following nutritional guidelines for dairy cattle [17]. The average values (± SD) were 32.3 ± 6.54 for daily milk yield (kg), 4.1 ± 0.36 for fat (%), and 3.7 ± 0.13 for protein (%). The cows had an average for days in milk (DIM) of 127.3 ± 60.22 (varying from 3 to 425) with a percentage of 55%, 42% and 3% at early, mid and late lactation, respectively. The percentages of 86%, 9%, and 5% were observed for 1^st^, 2^nd^, and from 3^rd^ to 5^th^ parity, respectively. Prior to collecting samples, a health assessment was performed, and any cows exhibiting clinical diseases or undergoing medical treatment were excluded from the study.

### Blood sampling

Blood samples were collected in 7 batches (i.e., sampling date): 3 batches in 2019 (300 cows) and 4 batches in 2020 (88 cows). Each cow was sampled once (*n* = 388). Five milliliters of blood from each cow were collected after the morning milking and before feeding through jugular venipuncture using vacutainer tubes containing 150 USP units of lithium heparin as an anticoagulant (Vacumed; FL Medical, Torreglia, Padua, Italy). All blood samples were maintained on ice until 2 h after blood sampling, followed by centrifugation at 3,500 × *g* for 1 min at 6 °C (Hettich Universal 16R Centrifuge), and then the plasma samples obtained were collected and stored at –20 °C until the analysis.

### Blood metabolic profile

Blood metabolites were analyzed using a clinical autoanalyzer (ILAB 650, Instrumentation Laboratory, Lexington, MA, USA) following methods proposed by Calamari et al. [18] and Hanasand et al. [19]. A complete metabolic profile was assessed covering energy-related metabolites (glucose, cholesterol, NEFA, BHB, urea, and creatinine), liver function/hepatic damage (aspartate aminotransferase [AST], γ-glutamyl transferase [GGT], total bilirubin [BILt], albumin, alkaline phosphatase [ALP], and paraoxonase [PON]), oxidative stress (total reactive oxygen metabolites [ROMt]; advanced oxidation protein products [AOPP]; ferric reducing antioxidant power [FRAP]; total thiol groups [SHp]), inflammation/innate immunity (CuCp, total proteins, globulins, haptoglobin, and myeloperoxidase), and minerals (Ca, P, Mg, Na, K, Cl and Zn). Kits from Instrumentation Laboratory (IL Test) were utilized to measure glucose, total proteins, albumin, haptoglobin, urea, Ca, AST, and GGT levels. Globulin concentration was estimated as the difference between total proteins and albumin, and potassium electrolytes (K^+^) were assessed using the potentiometer method (Ion Selective Electrode coupled to ILAB 600). Zn, NEFA, BHB, and CuCp were analyzed using the methods reported by Calamari et al. [18]. The concentrations of AOPP, ROMt, FRAP, and PON were determined according to Premi et al. [20].

### AfiLab equipment and near-infrared spectra storage

The AfiLab system is a spectrometer that uses a set of 32 discreet frequencies of light sources in the visible-NIR regimen (400–1,000 nm) based on light-emitting diodes as described by Schmilovitch et al. [21] and gives accurate estimates for fat in the range of 2% to 6% and for protein ranging from 2% to 5% (Afimilk, Israel, internal control) and for cheese-making traits [15]. During milking, the AfiLab system measures milk spectra on every 200 mL of milk flowing through the machine and reports an average of approximately 70 observations per cow in each milking session (~15 kg). Each observation is weighed with respect to its milk quantity (~0.20 to 0.33 mg). In addition, the AfiLab infrared information was zero-set calibrated once a month between the morning and afternoon milking sessions to eliminate possible bias as part of routine maintenance.

The AfiLab milk spectra and on-farm information from Afimilk system were collected concomitantly with the blood sampling. The AfiLab milk spectra were preprocessed considering the first derivative, estimated as the difference between consecutive NIR spectra information (${x}_{i}$) (${x}_{i}^{\prime }={x}_{i}-{x}_{i-1}$). The first derivative was then normalized using a Standard Normal Variate equation [${SVN}_{i}=({x}_{i}^{\prime }-{\stackrel{-}{x}}_{i}^{\prime })/{s}_{{x}_{i}^{\prime }}$], where ${x}_{i}^{\prime }$ is the first derivative of spectrum *i*, ${\stackrel{-}{x}}_{i}^{\prime }$ represent the mean of the first derivative for spectrum *i*, and ${s}_{{x}_{i}^{\prime }}=\sqrt{\frac{\sum ({x}_{i}^{\prime }-{\stackrel{-}{x}}_{i}^{\prime })^{2}}{m}}$ is the is the standard deviation for first derivative for spectrum *i* and *m* is the number of cows. The quality control of milk spectra was assessed by principal component analysis combined with Mahalanobis distance at a probability level < 0.05 [22]; after this data processing, four animals were removed from the analysis.

### Genomic data

All 388 cows were genotyped with the Geneseek Genomic Profiler Bovine 100k SNP Chip assay. The quality control was performed by removing the non-autosomal regions and autosomal SNP markers with a minor allele frequency of less than 0.05 and a significant deviation from Hardy–Weinberg equilibrium (*P* ≤ 10^−5^). Markers and samples with call rate lower than 0.95 were also removed. After spectra and genomic quality control, 380 cows with information for NIR AfiLab and 61,226 SNP markers remained in the dataset. Principal component analysis was used to assess population substructure based on the SNP markers using the ade4 R package [23] and no evidence of population stratification was found.

### Predictive ability

A 5-fold cross-validation (CV) scheme was used for assessing the predictive ability of the elastic-net (ENet) approach, which was chosen as the best-performing machine learning method in the blood metabolites prediction in previous studies of our group [9, 10]. We randomly split the dataset into five independent folds of approximately equal size. Thus, 4-fold were assigned to train the models and 1-fold to validate the model, and this CV procedure was repeated five times, predicting each fold in the validation set once. We used three elastic net (ENet) models to predict the target blood metabolite profile with increased complexity. The baseline model (M1) only considered NIR AfiLab information. In model 2 (M2), we combined NIR AfiLab and on-farm information (DIM and parity), while model 3 (M3) comprised NIR AfiLab, on-farm and genomic information.

### Elastic-net (ENet)

The ENet represents a penalized regression that combines LASSO (least absolute shrinkage and selection operator; ${l}_{1}= \sum _{w=1}^{p}\left|{\beta }_{w}\right|$) and RR (ridge regression; ${l}_{2}= \sum _{w=1}^{p}{\beta }_{w}^{2}$) regularization terms [24]. The ENet alpha parameter (α) controls the balance between the regularization terms ${l}_{1}$ and ${l}_{2}$, providing a balance between selection (LASSO) and shrinkage (RR) of the predictor variables effects. ENet is considered a robust approach when predictor variables have strong collinearity. The optimum weight values for λ and α in the ENet regression model are considered to reduce the loss function as follows:

$$L\left(\lambda , \alpha ,\beta \right)=min\left[ \sum _{i=1}^{N}\{{y}_{i}- \sum _{w=1}^{p}{x}_{iw}{\beta }_{w}{\}}^{2}+\lambda \left(\left(1-\alpha \right)\sum _{w=1}^{p}{\beta }_{w}^{2}+\alpha \sum _{w=1}^{p}\left|{\beta }_{w}\right|\right) \right],$$

where $N$ is the number of animals, $\alpha$ is a value between 0 (RR penalty) and 1 (LASSO penalty), and $\lambda$ is the regularization parameter that controls the amount of variable shrinkage. A random grid search was performed to find optimal values of α and λ ranging from 0.0 to 1.0 with an interval of 0.1 for each parameter. We implemented the ENet model using the glmnet R package [25]. The random search for α and λ was performed using the caret R package [26]. During the learning process of the ENet approach, the training set (4-fold) was split into an 80:20 ratio. The trained model with the highest accuracy and lowest mean square error (MSE) was then applied to a separate validation set (1-fold).

### Model performance

The predictive ability of the different models was assessed by Pearson’s correlation ($r=\text{cor}(y,\widehat{y})$) between observed phenotypes and predicted phenotypes ($\widehat{y}$). The predictive root mean squared error (RMSE) was $RMSE=\sqrt{\sum _{i=1}^{N}(y-\widehat{y}{)}^{2}/N}$, where *N* is the number of animals. The slope of the linear regression of $\widehat{y}$ on ${y}$ was also used to assess prediction bias. The relative difference (RD) in predictive ability was measured as $RD=\frac{(r_{\mathit m\mathit n}-r_{\mathit m1})}{r_{m1}}\times100$, where ${\textit{r}}_{\textit{m}1}$ is the predictive ability using the M1 approach and ${\textit{r}}_\textit{mn}$ is the predictive ability using the other models.

### Feature reduction prediction

The GWAS for blood metabolites were obtained with the following single-trait animal model via the genomic BLUP: 

$$\boldsymbol y=\boldsymbol X\boldsymbol b+\boldsymbol W\boldsymbol h+\boldsymbol Z\boldsymbol a+\boldsymbol e,$$

where $\boldsymbol{y}$ is a vector of blood metabolite information; **b** is the vector of fixed effects of days in milk with six classes (1: less than 60 d; 2: 60–120 d; 3: 121–180 d; 4: 181–240 d; 5: 241–300 d and 6: more than 300 d) and parity in 3 classes (1, 2, and ≥ 3 parities). The $\boldsymbol{h}$ and $\boldsymbol{a}$ are the random effects of batch and additive genetic effects, respectively; $\boldsymbol{X}$, $\boldsymbol{W}$, and $\boldsymbol{Z}$ are incidence matrices relating $\boldsymbol{y}$ to fixed effects ($\boldsymbol{b}$), batch effects ($\boldsymbol{h}$), and additive genomic breeding value ($\boldsymbol{a}$), respectively; and $\boldsymbol{e}$ is the residual effects.

The model was fitted under the following assumptions: $\boldsymbol{a}\sim\text{N}(0,\boldsymbol{G}{{\upsigma }}_{a}^{2})$, $\boldsymbol{h}\sim\text{N}(0,\boldsymbol{I}{{\upsigma }}_{\text{batch}}^{2})$ and $\boldsymbol{e}\sim\text{N}(0,\boldsymbol{I}{{\upsigma }}_{e}^{2})$, where $\sigma_e^{\text{2}}$, $\sigma_{batch}^2$, and $\sigma_e^2$ are variances for additive, batch, and residual effects, respectively; $\boldsymbol{I}$ is an identity matrix; and $\boldsymbol{G}$ is the genomic relationship matrix according to VanRaden [27]: $\boldsymbol{G}=\frac{{\boldsymbol{MM}}^{\prime }}{2\sum ^{{m}}_{j}=1 {p}_{j}\left(1-{p}_{j}\right)}$ where $\boldsymbol{M}$ is the SNP matrix with codes 0, 1, and 2 for genotypes *AA, AB*, and *BB*, adjusted for allele frequency, and ${\text{p}}_{\text{j}}$ is the frequency of the second allele of the $j$-th SNP.

The analyses were performed using the program blupf90+ [28]. The *P*-values were estimated by the SNP effects standardization as follows [29, 30]:

$$P\text{- value}=2 \left(1- {\upvarphi }\left(\frac{\left|{u}_{i}\right|}{{\sigma }_{{u}_{i}}}\right)\right)$$

where ${u}_{i}$ is the vector of the SNP marker effects, ${\sigma }_{{u}_{i}}$ is the standard deviation of SNP marker effects (${u}_{\text{i}}$) and ${\upvarphi }$ is the cumulative function of the normal distribution for the SNP effects standardization $\left(\frac{\left|{u}_{i}\right|}{{\sigma }_{{u}_{i}}}\right)$.

In order to evaluate the effectiveness of reducing dimensionality on predictive ability, we selected SNP markers from GWAS results performed in each training fold from 5-fold CV (i.e., 4-fold for training and 1-fold for validation) based on three thresholds of marker significance (${-\text{log}}_{10}\left(P\text{-value}\right)$) deemed as higher than 2.0, 2.5, and 3.0. The average number of SNP markers selected in each threshold is shown in Additional file 1: Table S2.

## Results

Descriptive statistics of the blood metabolic profile in the investigated population are reported in Additional file 1: Table S1. The cows enrolled in this study showed some relatively large data variability range for blood metabolites, which may indicate a low degree of physiological disturbance. Despite the absence of overt clinical disease, the high variability in certain blood biomarkers suggests the potential presence of subclinical conditions in specific individuals, which is expected in a large population. Specifically, we observed a degree of alteration in globulins (11% of cows > 50 g/L) and albumin (2% of cows < 30 g/L). Regarding urea levels, 43% of cows exceeded the threshold of ≥ 6.78 mmol/L. Less than 1% of the cows showed suspicion of hypomagnesemia or hypocalcemia, and less than 2% had hyperketonemia associated with high NEFA levels.

### Predictive performance of NIR data integrated with on-farm and genomic information

**Fig. 1:**
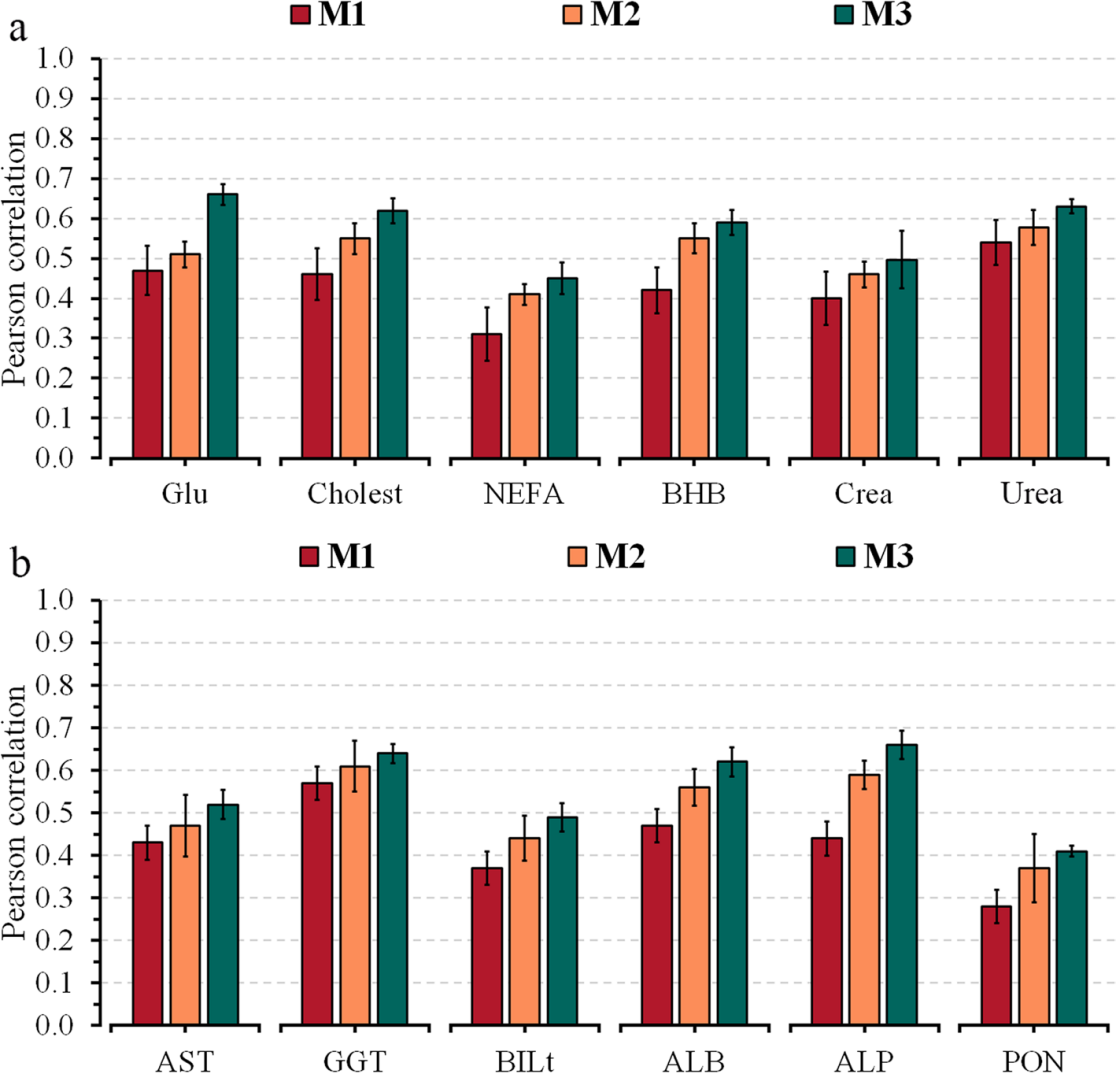
Predictive ability across 5-fold random cross-validation for Model 1 (NIR AfiLab data), Model 2 (NIR AfiLab data and on-farm), and Model 3 (NIR AfiLab data, on‑farm data, and genomic information) considering elastic net (ENet), for energy-related (**a**) and liver function and hepatic damage (**b**) blood metabolites. Data are shown as mean ± SD (black error bar line). Glu, glucose; Cholest, cholesterol; NEFA, non-esterified fatty acids; BHB, β-hydroxybutyrate; Crea, creatinine; AST, aspartate aminotransferase; GGT, γ-glutamyl transferase; BILt, total bilirubin; ALB, albumin; ALP, alkaline phosphatase; PON, paraoxonase

**Fig. 2:**
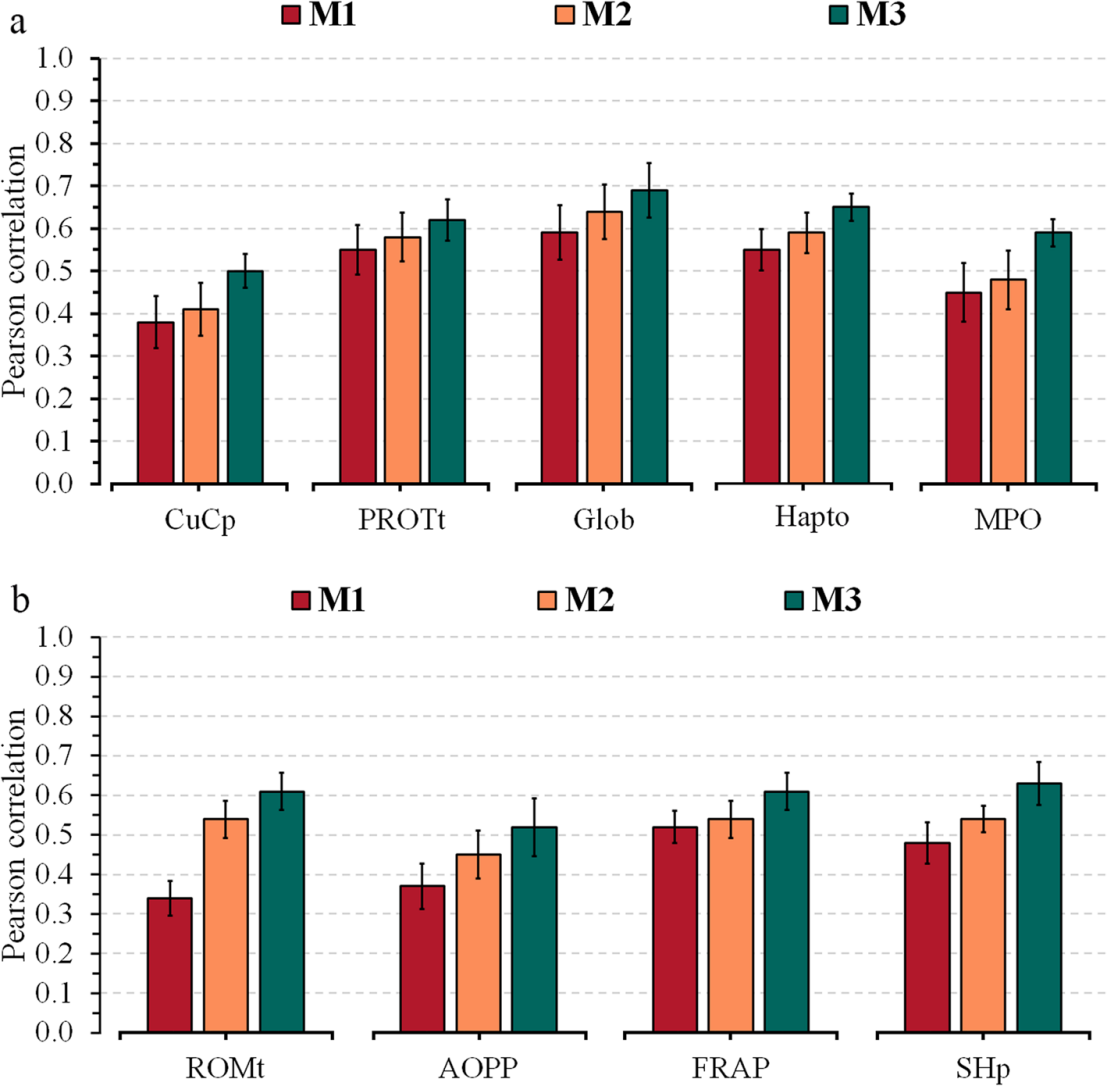
Predictive ability across 5-fold random cross-validation for Model 1 (NIR AfiLab data), Model 2 (NIR AfiLab data and on-farm), and Model 3 (NIR AfiLab data, on‑farm data, and genomic information) using the elastic net (ENet), for blood metabolites related to inflammation/innate immunity response (**a**) and oxidative stress (**b**).  Data are shown as mean ± SD (black error bar line). CuCp, ceruloplasmin; PROTt, total proteins; Glob, globulins; Hapto, haptoglobin; MPO, myeloperoxidase; ROMt, total reactive oxygen metabolites; AOPP, advanced oxidation protein products; FRAP, ferric reducing antioxidant power; SHp, total thiol groups

**Fig. 3:**
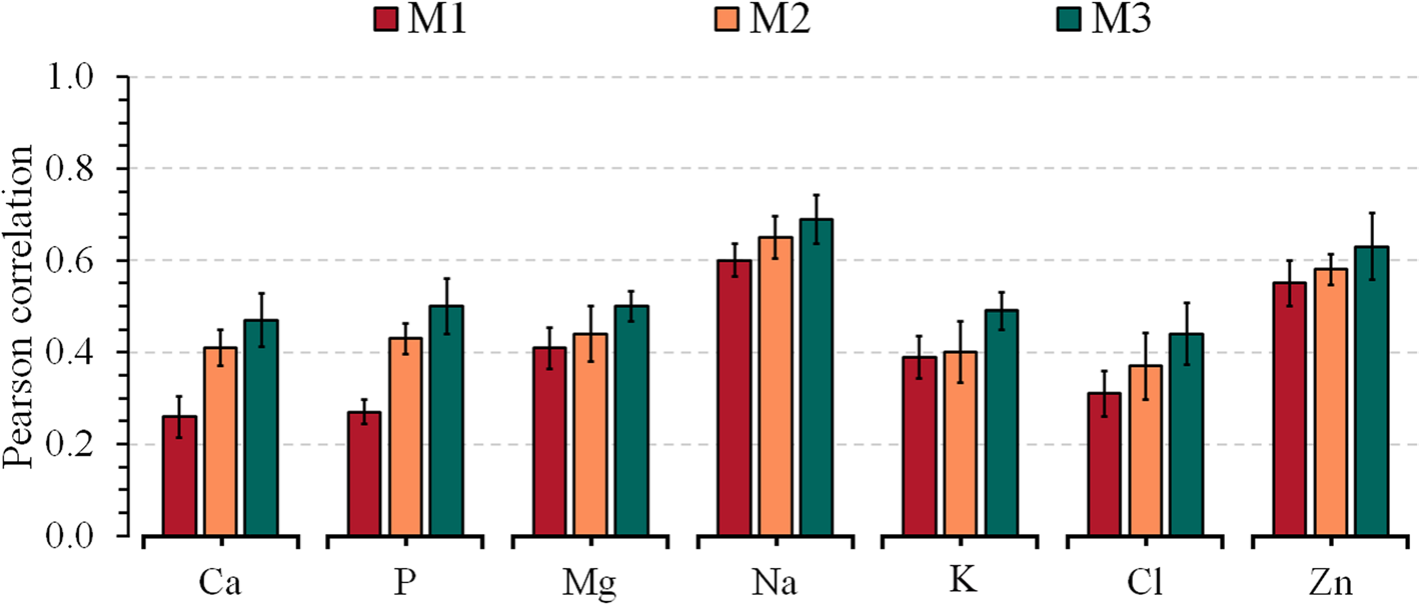
Predictive ability across 5-fold random cross-validation for Model 1 (NIR AfiLab data), Model 2 (NIR AfiLab data and on-farm), and Model 3 (NIR AfiLab data, on‑farm data, and genomic information) using the elastic net (ENet) for blood minerals.  Data are shown as mean ± SD (black error bar line). Traits: Ca, calcium; P, phosphorus; Mg, magnesium; Na, sodium; K, potassium; Cl, chlorine and Zn, zinc

Model M1, which included only the milk NIR information, achieved the lowest predictive ability ($r$) compared to the models including also on-farm data (M2) and both on-farm and genomic information (M3). For M1, the $r$-values ranged from 0.31 to 0.54 for energy-related metabolites (Fig. 1a), 0.28 to 0.57 for liver function/hepatic damage (Fig. 1b), 0.38 to 0.59 for inflammation/innate immunity (Fig. 2a), 0.34 to 0.52 for oxidative stress metabolites (Fig. 2b), and from 0.26 to 0.60 for minerals (Fig. 3) (see Additional file 1: Tables S3–S5, respectively). The combination of NIR and information on the farm (M2) achieved an average increase of 19% (3%–59%) in relation to the M1 model, with *r*-values ranging from 0.41 to 0.58 for energy-related metabolites, 0.37 to 0.61 for liver function/hepatic damage, 0.41 to 0.64 for inflammation/innate immunity, 0.45 to 0.54 for oxidative stress metabolites, and from 0.37 to 0.68 for minerals. Integrating on-farm and genomic information into NIR (M3) resulted in a 39% (12%–85%) average increase of *r* compared to M1, with *r*-values varying from 0.45 to 0.66 for energy-related metabolites, 0.41 to 0.66 for liver function/hepatic damage, 0.50 to 0.69 for inflammation/innate immunity, 0.52 to 0.63 for oxidative stress metabolites, and from 0.44 to 0.69 for minerals.

The results obtained from the M2 and M3 with a 5-fold CV show that including on-farm information (DIM and parity) or on-farm and genomic information enhances the predictive ability of NIR infrared prediction (see Additional file 2: Fig. S2). Moreover, the use of on-farm information in NIR infrared predictions improves the predictive ability by 3%–59%, with significant improvements seen for P, ROMt, Ca, ALP, NEFA, PON, and BHB (see Additional file 2: Fig. S2). When both on-farm and genomic information are combined, the *r*-value increases from 12% to 85%, with an increase of more than 30% in 16 metabolites (P, Ca, ROMt, ALP, PON, NEFA, Cl, AOPP, BHB, glucose, cholesterol, BILt, albumin, CuCp, SHp, and myeloperoxidase) out of the 28 evaluated. The slope coefficients obtained from M2 and M3 indicate that the predictions were slightly underestimated or overestimated. The slope values for M2 ranged from 0.94 to 1.07, while for M3, the values were between 0.95 and 1.05. Model M1 showed more bias, with values varying from 0.85 to 1.29 (Additional file 2: Tables S3–S5).

### Impact of feature selection on NIR AfiLab prediction performance

**Fig. 4:**
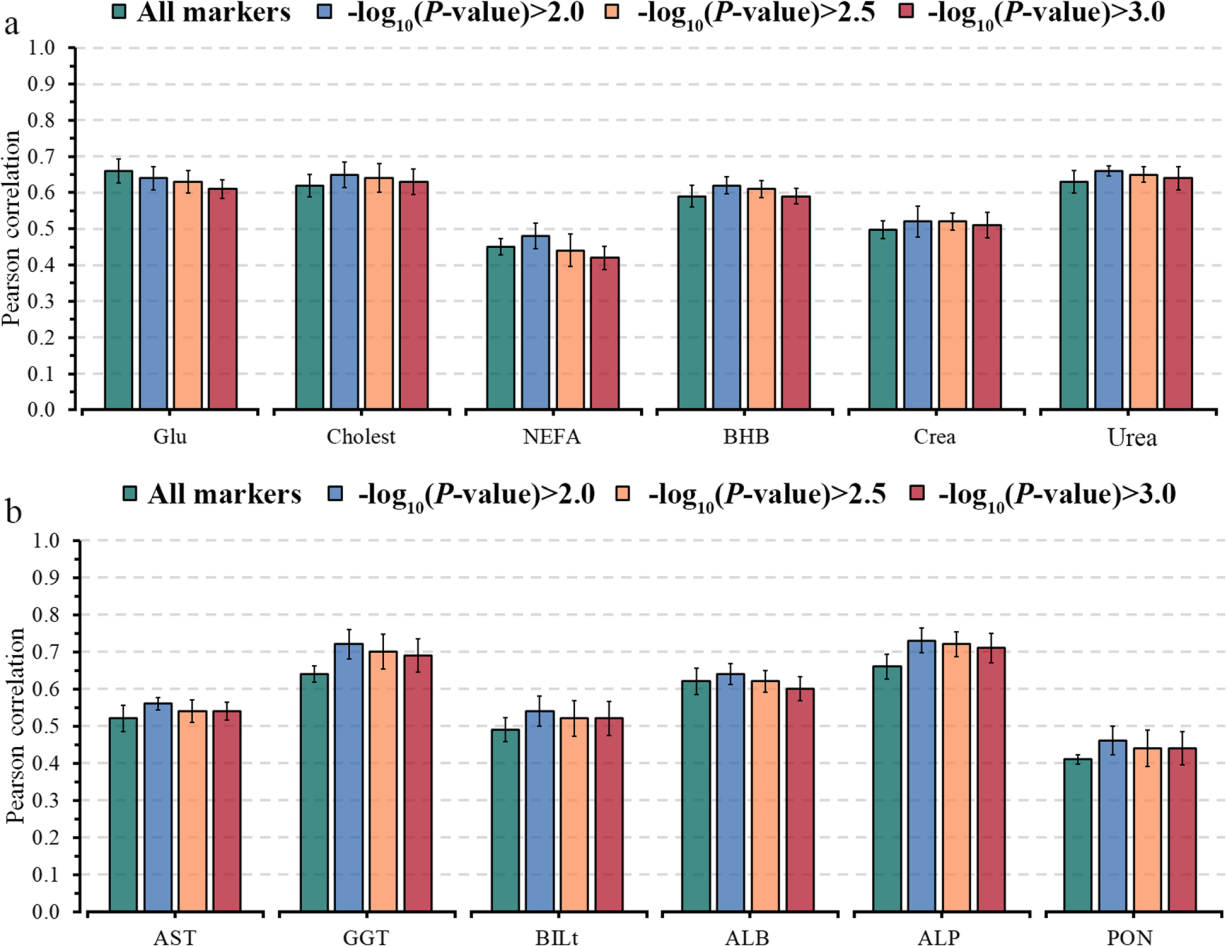
Predictive ability, including standard errors, for energy-related (**a**) and liver function/hepatic damage (**b**) blood metabolites for ENet fitting all markers and using three thresholds based on marker significance: ${-\log}_{10}(P\text{-value})>2.0$**,** ${-\log}_{10}(P\text{-value})>2.5$ and ${-\log}_{10}(P\text{-value})>3.0$. Traits: Glu, glucose; Cholest, cholesterol; NEFA, non-esterified fatty acids; BHB, β-hydroxybutyrate; Crea, creatinine; AST, aspartate aminotransferase; GGT, γ-glutamyl transferase; BILt, total bilirubin; ALB, albumin; ALP, alkaline phosphatase; PON, paraoxonase

**Fig. 5:**
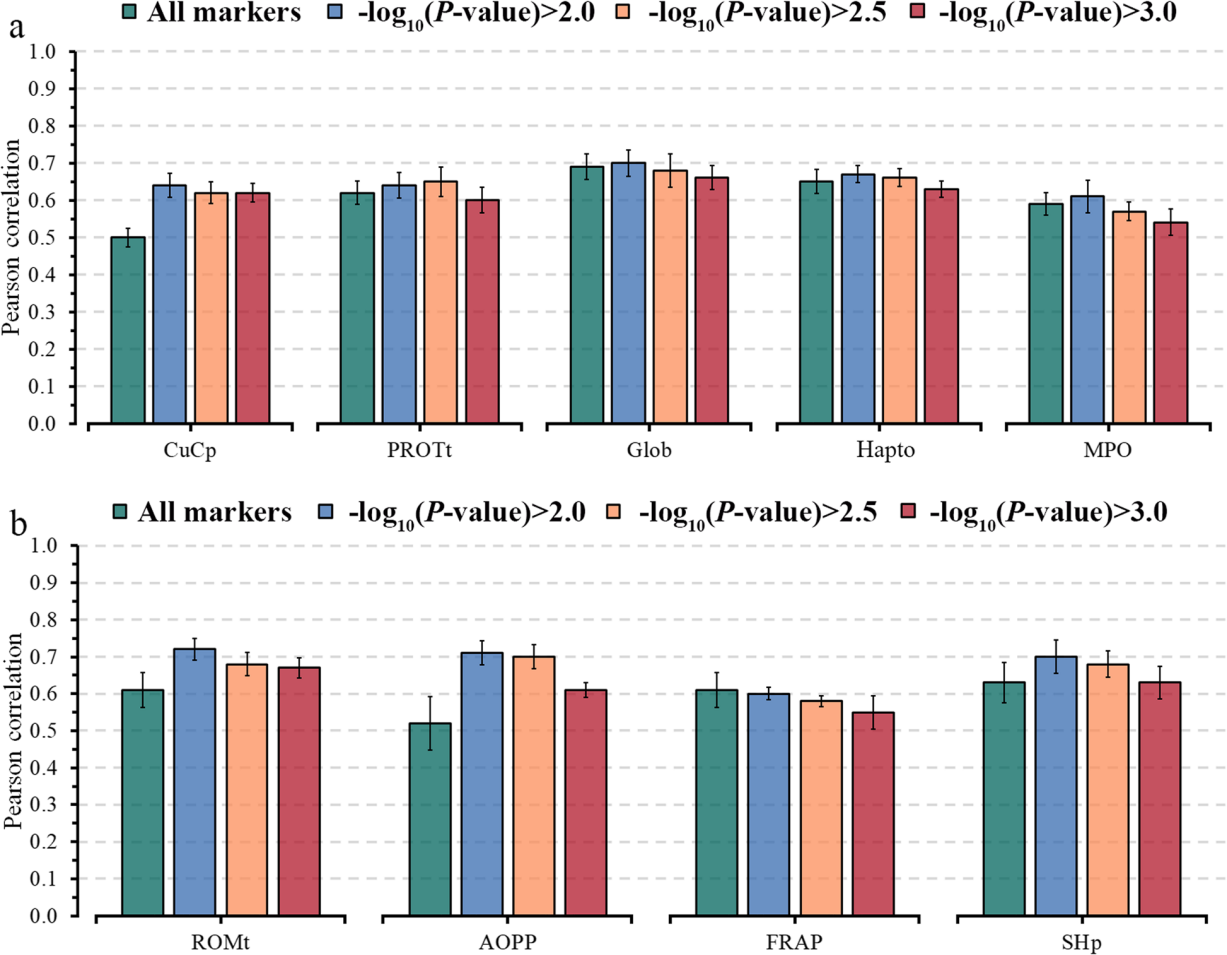
Predictive ability, including standard errors, for inflammation/innate immunity (**a**) and oxidative stress (**b**) blood metabolites for ENet fitting all markers and using three thresholds based on marker significance: ${-\log}_{10}(P\text{-value})>2.0$, ${-\log}_{10}(P\text{-value})>2.5$ and ${-\log}_{10}(P\text{-value})>3.0$. Traits: CuCp, ceruloplasmin; PROTt, total proteins; Glob, globulins; Hapto, haptoglobin; MPO, myeloperoxidase; ROMt, total reactive oxygen metabolites; AOPP, advanced oxidation protein products; FRAP, ferric reducing antioxidant power; SHp, total thiol groups

**Fig. 6:**
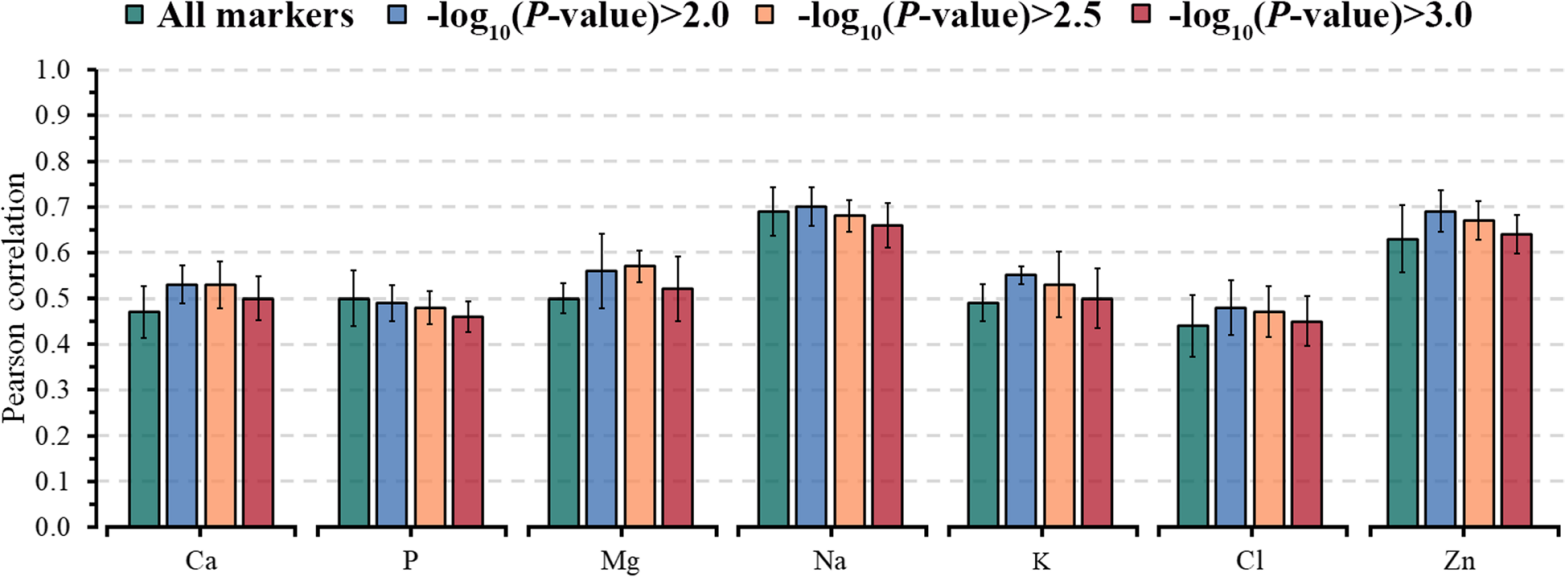
Predictive ability, including standard errors, for blood minerals for ENet fitting all markers and using three thresholds based on marker significance: ${-\log}_{10}(P\text{-value})>2.0$, ${-\log}_{10}(P\text{-value})>2.5$ and ${-\log}_{10}(P\text{-value})>3.0$. Traits: Ca, calcium; P, phosphorus; Mg, magnesium; Na, sodium; K, potassium; Cl, chlorine and Zn, zinc

Using selected markers based on GWAS analyses improved the predictive ability when applying a threshold of ${-\text{log}}_{10}\left(P\text{-value}\right)$, except for Glu, FRAP, and P. The predictive ability (*r*) varied from 0.48 to 0.66 for energy-related metabolites, 0.46 to 0.73 for liver function/hepatic damage, 0.61 to 0.70 for inflammation/innate immunity, 0.60 to 0.72 for oxidative stress metabolites, and 0.48 to 0.70 (Figs. 4, 5, 6). On average, preselecting markers with a threshold of ${-\text{log}}_{10}\left(P\text{-value}\right)>2$ predictions achieved higher gains for oxidative stress metabolites (RD = 16%, ranging from −2% to 36%) and for liver function/hepatic damage traits (RD = 9%, ranging from 3% to 12%), while lower gain was observed for energy-related metabolites (RD = 4%, ranging from −3% to 7%).

The predictive ability of the model M3, considering selected markers with a threshold of ${-\text{log}}_{10}\left(P\text{-value}\right)$ > 2.5 showed slight improvements in the predictive ability compared to the threshold of 2. The threshold of 2.5 resulted in an average improvement of 8.8% in the *r-*value for 20 out of 28 evaluated metabolites (Figs. 4, 5, 6). However, using a more restrictive threshold (${-\text{log}}_{10}\left(P\text{-value}\right)$> 3) to preselect markers led to a slight gain or reduction in predictive ability compared to using all markers in M3 (Figs. 4, 5, 6). AOPP and CuCp showed an RD higher than 10% for all evaluated thresholds, indicating that few genetic markers can explain their variability.

## Discussion

### Predictive performance integrating on-farm and genetic markers in NIR AfiLab

The study’s objective was to evaluate the potential of integrating the AfiLab NIR milk analyzer with on-farm data (DIM and parity) and genetic marker information for the prediction of blood metabolites in Holstein cows. The Fourier-transform mid-infrared (FTIR) technique has become a broadly explored tool to predict complex traits, such as the blood metabolic profile in dairy cattle [8, 9, 31, 32]. Although in-line NIR infrared showed low to moderate predictive ability for blood metabolites (Figs. 1, 2, 3), it represents an alternative for daily monitoring at the herd level due to its daily availability. Previous studies have pointed out that using an integration of infrared with on-farm information (e.g., DIM, parity, and behavior parameters) or with on-farm and genetic markers allows improvement in infrared predictive ability for both FTIR [33–35] and NIR [15].

The adoption of the multi-data integration approach for predicting complex phenotypes is on the rise, primarily because it offers a more precise representation of the intricate biological architecture associated with traits. Creating a training dataset structure from different sources on a massive is an integration and data architecture challenge. These data have different structure, dimensionality, resolution, and integrity. Such data can be used to build predictive models that accurately predict the unknown target traits in different farms and seasons. High-throughput technologies can gather on-farm data using automated in-line sensors installed in milking parlors. These sensors assess milk quality and quantity, recording information related to various aspects of individuals, including DIM, parity, and physiological parameters (e.g., respiratory rate and rumination time). The data obtained by these sensors can be combined with NIR information to predict novel phenotypes, which can then be used for selective breeding and management purposes. In recent years, there has been a growing emphasis on collecting on-farm data and integrating it to predict economically significant phenotypic traits in dairy cattle [8, 15, 33]. In this study, we observed that including on-farm information (DIM and parity) in the NIR predictions (M2) resulted in improvements on $r$-values with an average RD of 19% for energy-related metabolites, 20% for liver function/hepatic damage metabolites, 7% for inflammation/innate immunity metabolites, 24% for oxidative stress metabolites, and 23% for minerals (see Additional file 2: Fig. S1).

The increased *r*-value could be attributed to the direct influence of the lactation period on energy requirements and changes in milk yield, as well as milk fat and protein concentrations [36]. Since metabolic disorders also directly impact milk yield and quality [37], separating the effects of on-farm factors from those caused by metabolic distress can improve the predictive power of statistical algorithms [8]. In this context, Wu et al. [38] found that on-farm information (DIM and parity) has a great influence on variations in serum biochemical parameters and hormones related to protein status, energy supply, liver and kidney function, and oxidative stress of mid-lactation Holstein cows. Thus, by combining DIM and parity with the NIR infrared predictions, we reduced the prediction error in the independent population by accounting for varied physiological conditions along the lactation curve. Furthermore, integrating on-farm explanatory variables allows for capturing the lactation stage that explains the variability of the target phenotype, thus enhancing the accuracy of the NIR predictions.

When predicting blood metabolites using the M2, FRAP, and SHp demonstrated a better predictive ability on average inflammation/innate immunity metabolites, *r-*values ranged from 0.41 for CuCp to 0.64 for globulins and oxidative stress metabolites with *r*-values of 0.45 for AOPP and 0.54 for ROMt. These values were similar to those achieved using NIR spectra [10] but lower when compared to FTIR spectra [8, 9, 39]. A continuous exchange between blood and milk occurs through the blood-milk barrier, leading to a good predictive ability for inflammation and innate immunity-related metabolites because milk contains proteins like CuCp and haptoglobin originating from the bloodstream. In addition, during mammary gland inflammatory processes, the acute-phase proteins are also directly produced by milk leukocytes and mammary epithelial cells [40–42].

Myeloperoxidase, an enzyme released by activated neutrophils during inflammatory responses, is also found in milk and is associated with ongoing infections [40, 43]. On the other hand, oxidative stress has been associated with metabolic disorders in dairy cows, and milk contains measurable plasma thiols, reactive oxygen metabolites, and AOPP, which can indicate the individual’s oxidative stress status [44]. In this context, our results demonstrate that using NIR infrared with on-farm data can achieve moderate accuracy in predicting blood oxidant-antioxidant status. This allows the identification of cows with high oxidative stress imbalance and detect energetic and metabolic impairments.

Mota et al. [8] observed improved prediction of blood traits by integrating milk FTIR spectra with on-farm and genetic markers, especially for metabolites under strong genetic control, i.e., higher heritability. Our results suggest that blending on-farm and genomic data in NIR AfiLab prediction can help predict variations in blood metabolites. In particular, we observed an average increase of 34% in the *r-*value for the investigated metabolites compared with M1 and 13% with M2 (see Additional file 2: Fig. S1). Increasing the power of prediction methods is an area of active research that aims to enable more efficient identification and allocation of cows less prone to be affected by metabolic disorders. Data integration could help predict the evolution of the metabolic response in the medium and long term and understand whether the animals are in a phase of adaptation or chronic stress [20].

### Phenotype prediction using marker selection on NIR AfiLab prediction performance

The standard procedure for using genetic markers for infrared predictions is to use all information obtained from the SNP array as predictors. However, several complex biological downstream processes affect the phenotypic variability, and using SNP predictors more closely linked to the true quantitative trait loci (QTL) affecting the target phenotypes may increase the NIR AfiLab prediction performance. Selecting optimal markers based on their significance for the target trait is a crucial step in reducing the dimensionality of information in predictive models when multiple sources of information are combined. This helps to minimize the number of parameters in the model, avoiding overfitting and potentially improving the accuracy of predictions. The improvements in the predictive ability of a selected subset of markers depend on how well it matches the genetics underlying the phenotypic trait(s), and with a sufficient number of markers able to capture the trait variability (see Additional file 1: Table S2 and Additional file 2: Fig. S2–S4).

Previous studies adopted different strategies to preselect predictors by directly excluding uninformative markers via machine learning [45–47] or assigning weights to markers according to their contributions to trait variability [48]. Piles et al. [47] and Li et al. [49] showed that feature selection strategies improved the predictive ability of complex traits. We observed that preselected markers using a less conservative threshold (${-\text{log}}_{10}\left(P\text{-value}\right)$ > 2.0) led to improvements in the *r*-value from 10% to 36%, even if a reduction was observed for glucose (3%) and FRAP (2%). The notable improvement in predictive capability seen with CuCp and AOPP can be attributed to the advantage gained from utilizing the most influential SNPs that bear biological relevance to the target trait (Additional file 2: Fig. S6 and S8). This is especially pronounced in traits influenced by QTL, which has a relatively significant effect (Additional file 2: Fig. S5–S9). Fragomeni et al. [50] and Mancin et al. [45] highlighted the advantages of removing non-informative SNP, where better accuracy was achieved by constructing the ***G*** matrix by considering the window region where the QTL was identified or by using only QTL information.

Selected SNPs have also been observed to capture significant within-family variation and Mendelian segregation effects [51]. Our findings emphasized that combining NIR infrared and on-farm data with selected markers significantly associated (${-\text{log}}_{10}\left(P\text{-value}\right)$ > 2.0) with the target trait increased the predictive ability for predicting blood metabolites in dairy cattle (see Additional file 2: Fig. S2–S4). On the other hand, when dealing with more complex traits (i.e., polygenic traits), combining NIR infrared and on-farm information with approximately 5k selected markers (see Additional file 1: Table S2) resulted in a decrease in predictive ability compared to using all markers for glucose, NEFA, albumin, myeloperoxidase, FRAP, P and Na (see Additional file 2: Fig. S2–S4). These reductions were more remarkable as the selection criteria were more restrictive, i.e., ${-\text{log}}_{10}\left(P\text{-value}\right)$ > 2.5 and 3.0 (see Additional file 2: Fig. S2–S4), and this could be due to reduced linkage disequilibrium between the SNP and the true QTL [52].

Given this, comparing less restrictive threshold (> 2) to more restrictive (> 2.5 and > 3) showed predictive abilities that were lower by about 2% and 5% for energy-related metabolites, 3% and 4% for liver function/hepatic damage metabolites, 2% and 6% for inflammation/innate immunity metabolites, 3% and 10% for oxidative stress metabolites, and 2% and 7% for minerals. This result highlights the importance of preselecting markers for predicting complex phenotypes depending on how much this dimension reduction accurately selects predictor variables related to the target trait. Hence, our findings indicate that combining NIR infrared, on-farm and genomic information, or selected markers from GWAS, considering a threshold of ${-\text{log}}_{10}\left(P\text{-value}\right)$ > 2.0 can enhance the predictive ability of metabolic imbalances in dairy cattle. As such, using multi-layer information to predict blood metabolites at the herd level in a rapid, affordable, and real-time manner unveils the promising potential of milk NIR spectra predictions in the early detection of metabolic disorders. Additionally, the outcomes of our study reveal moderate to high predictive abilities, making the prediction equations potentially useful in guiding herd management especially for its ability to capture day-by-day fluctuations, and formulating breeding recommendations for more resilient cows.

## Conclusions

Integrating NIR spectra with on-farm and genomic information yielded a better predictive ability for blood metabolites than the model that relied solely on AfiLab milk NIR spectra in Holstein cattle. Indeed, the combination of NIR spectral data with on-farm and genomic information consistently outperformed prediction based on NIR spectra by an average of 34%. Preselecting genetic markers from GWAS has been shown to be an efficient strategy for dimensionality reduction by selecting trait-relevant markers, improving predictive ability because it extracts a smaller number of informative markers. We showed that preselecting genetic markers with a less restrictive threshold (${-\text{log}}_{10}\left(P\text{-value}\right)>2.0$) resulted in better performance than considering all markers. Additionally, we found that using more restrictive thresholds (${-\text{log}}_{10}\left(P\text{-value}\right)$ > 2.5 and 3.0) led to a negligible improvement in the predictive ability of blood metabolites.

### Supplementary Information

**Additional file 1:** **Table S1.** Descriptive statistics for blood metabolites in Holstein cows. **Table S2.** Average number of SNP markers selected for each training fold used during the cross-validation performance considering 5-fold. **Table S3.** Average prediction performance (± SD) of milk AfiLab NIR alone (model 1, M1), considering the systematic effect of days in milk and parity (model 2, M2) and considering the systematic effect of days in milk, parity, and genomic information (model 3, M3) for the 5-fold random cross‑validation scenario using the elastic net method for energy-related and liver function/hepatic damage blood metabolites. **Table S4.** Average prediction performance (± SD) of milk AfiLab NIR alone (model 1, M1), considering the systematic effect of days in milk and parity (model 2, M2) and considering the systematic effect of days in milk, parity, and genomic information (model 3, M3) for the 5-fold random cross‑validation scenario using the elastic net method for inflammation/innate immunity response and oxidative stress metabolites. **Table S5.** Average prediction performance (± SD) of milk AfiLab NIR alone (model 1, M1), considering the systematic effect of days in milk and parity (model 2, M2) and considering the systematic effect of days in milk, parity, and genomic information (model 3, M3) for the 5-fold random cross‑validation scenario using the elastic net method for blood minerals.

**Additional file 2:** **Fig. S1.** Relative difference (%) in predictive ability for 5-fold random cross-validation scenarios using Elastic-net for Model 2 (M2; milk NIR data and on-farm data) and Model 3 (M3; milk NIR data, on-farm and genomic information) against Model 1, which considers only the NIR infrared data. Data are shown as mean ± SD (red error bar line). Glu – glucose; Cholest – cholesterol; NEFA – non-esterified fatty acids; BHB – β-hydroxybutyrate; Crea – creatinine; AST – aspartate aminotransferase; GGT – γ-glutamyl transferase; BILt – total bilirubin; ALB – albumin; ALP – alkaline phosphatase; PON – paraoxonase; CuCp – ceruloplasmin; Glob – globulins; PROTt – total proteins; Hapto – haptoglobin; MPO – myeloperoxidase; ROMt – total reactive oxygen metabolites; AOPP – advanced oxidation protein products; FRAP – ferric reducing antioxidant power; SHp – total thiol groups; Ca – calcium; P – phosphorus; Mg – magnesium; K – potassium; Na – sodium; Cl – chlorine; Zn – zinc. **Fig. S2.** Relative gain in predictive ability Pearson correlation, considering three thresholds based on marker significance (-log_10_(*P*-value)) higher than 2.0, 2.5, and 3.0 against fitting all 61k SNPs, including standard errors, assessed for energy-related (**a**) and liver function and hepatic damage (**b**) blood metabolites. Data are shown as mean ± SD (black error bar line). Glu – glucose; Cholest – cholesterol; NEFA – non-esterified fatty acids; BHB – β-hydroxybutyrate; Crea – creatinine; AST – aspartate aminotransferase; GGT – γ-glutamyl transferase; BILt – total bilirubin; ALB – albumin; ALP – alkaline phosphatase; PON – paraoxonase. **Fig. S3.** Relative gain in predictive ability Pearson correlation, considering three thresholds based on marker significance (-log_10_(*P*-value)) higher than 2.0, 2.5 and 3.0 against fitting all 61k SNPs, including standard errors, assessed for inflammation/innate immunity response (**a**) and oxidative stress blood metabolites (**b**). Data are shown as mean ± SD (black error bar line). CuCp – ceruloplasmin; PROTt – total proteins; Glob – globulins; Hapto – haptoglobin; MPO – myeloperoxidase; ROMt – total reactive oxygen metabolites; AOPP – advanced oxidation protein products; FRAP – ferric reducing antioxidant power; SHp – total thiol groups. **Fig. S4.** Relative gain in predictive ability Pearson correlation, considering three thresholds based on marker significance (higher than 2.0, 2.5 and 3.0 against fitting all 61k SNPs, including standard errors, assessed for blood minerals. Data are shown as mean ± SD (black error bar line). Ca – calcium; P – phosphorus; Mg – magnesium; Na – sodium; K – potassium; Cl – chlorine; Zn – zinc. **Fig. S5.** Manhattan plot for the average value of markers significance (-log_10_(*P*-value)) across the 5-fold cross-validation for energy-related metabolites. Glu – glucose; Cholest – cholesterol; NEFA – non-esterified fatty acids; BHB – β-hydroxybutyrate; Crea – creatinine. **Fig. S6.** Manhattan plot for the average value of markers significance (-log_10_(*P*-value)) across the 5-fold cross-validation for blood metabolites related to inflammation/innate immunity response. CuCp – ceruloplasmin; PROTt – total proteins; Glob – globulins; Hapto – haptoglobin; MPO – myeloperoxidase. **Fig. S7.** Manhattan plot for the average value of markers significance (-log_10_(P-value)) across the 5-fold cross-validation for blood metabolites related to liver function and hepatic damage. AST – aspartate aminotransferase; GGT – γ-glutamyl transferase; BILt – total bilirubin; ALB – albumin; ALP – alkaline phosphatase; PON – paraoxonase. **Fig. S8.** Manhattan plot for the average value of markers significance (-log_10_(*P*-value)) across the 5-fold cross-validation for oxidative stress blood metabolites. ROMt – total reactive oxygen metabolites; AOPP – advanced oxidation protein products; FRAP – ferric reducing antioxidant power; SHp – total thiol groups. **Fig. S9.** Manhattan plot for the average value of markers significance (-log_10_(*P*-value)) across the 5-fold cross-validation for blood minerals. Ca – calcium; P – phosphorus; Mg – magnesium; K – potassium; Na – sodium; Cl – chlorine; Zn – zinc.

## Data Availability

The phenotypic and genotypic information are available for academic use from the authors upon reasonable request. The spectral data that support the findings of this study are deposited with Afimilk Ltd., and access is restricted as they were used under license for the current study and are therefore not publicly available. However, they can be obtained from the authors upon reasonable request and with the permission of Afimilk Ltd.

## Abbreviations

ALP: Alkaline phosphatase
AOPP: Advanced oxidation protein products
AST: Aspartate aminotransferase
BHB: β-Hydroxybutyrate
BILt: Total bilirubin
CuCp: Ceruloplasmin
CV: Cross–validation
DIM: Days in milk
ENet: Elastic net
FRAP: Ferric reducing antioxidant power
FTIR: Fourier transform mid infrared
GGT: γ-Glutamyl transferase
GWAS: Genome wide association study
LASSO: Least absolute shrinkage and selection operator
M1: Base model
M2: Model 2
M3: Model 3
NEFA: Non–esterified fatty acids
NIR: Near infrared
PON: Paraoxonase
QTL: Quantitative trait loci
*r*: Coefficient of correlation
RD: Relative difference
RMSE: Root mean squared error
ROMt: Total reactive oxygen metabolites
RR: Ridge regression
SHp: Total thiol group
SNP: Single nucleotide polymorphism
