# Supplementary material for: Combining genetic markers, on-farm information and infrared data for the in-line prediction of blood biomarkers of metabolic disorders in Holstein cattle
Source: J Anim Sci Biotechnol. 2024 Jun 9;15:83. doi: 10.1186/s40104-024-01042-3 (PMC11162571; doi:10.1186/s40104-024-01042-3)
Supplement: Supplementary file 1 — Additional file 1: Table S1. Descriptive statistics for blood metabolites in Holstein cows. Table S2. Average number of SNP markers selected for each training fold used during the cross-validation performance considering 5-fold. Table S3. Average prediction performance (± SD) of milk AfiLab NIR alone (model 1, M1), considering the systematic effect of days in milk and parity (model 2, M2) and considering the systematic effect of days in milk, parity, and genomic information (model 3, M3) for the 5-fold random cross‑validation scenario using the elastic net method for energy-related and liver function/hepatic damage blood metabolites. Table S4. Average prediction performance (± SD) of milk AfiLab NIR alone (model 1, M1), considering the systematic effect of days in milk and parity (model 2, M2) and considering the systematic effect of days in milk, parity, and genomic information (model 3, M3) for the 5-fold random cross‑validation scenario using the elastic net method for inflammation/innate immunity response and oxidative stress metabolites. Table S5. Average prediction performance (± SD) of milk AfiLab NIR alone (model 1, M1), considering the systematic effect of days in milk and parity (model 2, M2) and considering the systematic effect of days in milk, parity, and genomic information (model 3, M3) for the 5-fold random cross‑validation scenario using the elastic net method for blood minerals. [file 40104_2024_1042_MOESM1_ESM.pdf]

Additional file 1

Table S1 – Descriptive statistics for blood metabolites in Holstein cows.

Blood metabolites[1]	n	mean	SD	CV	$Q_{1\%}$	$Q_{99\%}$
Glucose, mmol/l	380	4.47	0.302	7%	3.74	5.27
Cholesterol, mmol/l	379	4.96	1.227	25%	2.04	7.79
NEFA, mmol/l	379	0.14	0.173	127%	0.04	0.54
BHB, mmol/l	380	0.52	0.203	39%	0.25	1.29
Urea, mmol/l	380	6.52	1.029	16%	4.09	9.08
Creatinine, µmol/l	380	80.99	5.701	7%	71.75	93.44
AST, U/l	380	99.62	23.829	24%	68.34	175.69
GGT, U/l	380	26.67	8.405	32%	14.63	52.94
BILt, µmol/l	380	2.20	1.147	52%	0.56	6.61
Albumin, g/l	380	37.02	2.287	6%	29.18	41.23
ALP, U/l	380	66.38	19.505	29%	33.44	124.91
PON, U/ml	380	104.85	19.279	18%	58.17	154.54
Ceruloplasmin, µmol/l	380	1.78	0.605	34%	0.76	3.76
PROTt, g/l	380	81.10	4.957	6%	71.70	95.09
Globulins, g/l	380	44.08	5.461	12%	36.13	60.97
Haptoglobin, g/l	380	0.38	0.331	87%	0.11	1.50
Myeloperoxidase, U/l	380	460.61	72.988	16%	284.32	672.20
ROMt, gH_2O_2/100ml	380	12.64	3.202	25%	5.89	23.25
AOPP, µmol/l	380	48.44	9.247	19%	29.66	72.68
FRAP, µmol/l	379	209.91	60.878	29%	126.78	315.00
SHp, µmol/l	357	389.75	51.296	13%	271.48	515.88
Calcium, mmol/l	380	2.53	0.116	5%	2.21	2.78
Phosphorus, mmol/l	380	2.01	0.332	17%	1.27	2.82
Magnesium, mmol/l	380	1.01	0.103	10%	0.74	1.24
Sodium, mmol/l	380	143.31	2.740	2%	135.86	148.43
Potassium, mmol/l	380	4.22	0.354	8%	3.50	5.22
Chlorine, mmol/l	380	102.96	2.235	2%	97.06	109.83
Zinc, µmol/l	380	11.45	2.218	19%	6.48	17.14

[1]NEFA - non-esterified fatty acids; BHB - β-hydroxybutyrate; AST - aspartate aminotransferase; GGT - γ -glutamyl transferase; BILt - total bilirubin; ALP - alkaline phosphatase; PON - paraoxonase; ROMt - total reactive oxygen metabolites; AOPP - advanced oxidation protein products; FRAP - ferric reducing antioxidant power; SHp – total thiol groups; PROTt - total proteins.

N: number of records; SD: standard deviation; $Q_{1\%}$ and $Q_{99\%}$ represent 1 and 99% of the trait quantile in percentage.

Table S2 – Average number of SNP markers selected for each training fold used during the cross-validation performance considering 5-fold.

Trait	Number of SNP based on -$\log_{10}$(p-value)		
Energy-related metabolites	> 2.0	> 2.5	> 3.0
Glucose, mmol/l	3436 ± 17.34	1974 ± 6.57	1112 ± 2.43
Cholesterol, mmol/l	7774 ± 39.08	4645 ± 24.27	2761 ± 12.72
BHB, mmol/l	5044 ± 32.08	2680 ± 15.82	1407 ± 7.65
NEFA, mmol/l	2652 ± 21.86	1108 ± 8.68	517 ± 3.07
Urea, mmol/l	7192 ± 36.10	4234 ± 26.98	2478 ± 19.46
Creatinine, µmol/l	4833 ± 30.35	2454 ± 21.26	1270 ± 11.31
Liver function/hepatic damage			
Albumin, g/l	4406 ± 23.67	2280 ± 11.75	1197 ± 6.37
ALP, U/l	8601 ± 33.86	5377 ± 22.20	3367 ± 14.54
AST, U/l	4643 ± 28.50	2508 ± 13.18	1324 ± 5.99
BILt, µmol/l	4852 ± 31.4	2559 ± 23.35	1318 ± 9.88
GGT, U/l	5512 ± 25.82	2943 ± 15.95	1591 ± 5.68
PON, U/ml	6892 ± 43.95	3939 ± 33.6	2306 ± 17.93
Inflammation/innate immunity			
Ceruloplasmin, µmol/l	5584 ± 6.15	3045 ± 3.61	1734 ± 4.02
Haptoglobin, g/l	2937 ± 21.64	1324 ± 8.8	608 ± 3.38
PROTt, g/l	5654 ± 17.49	3139 ± 11.49	1758 ± 5.76
Globulins, g/l	4776 ± 23.76	2534 ± 12.02	1376 ± 5.65
Myeloperoxidase, U/l	4935 ± 17.31	2709 ± 9.67	1442 ± 6.59
Oxidative stress metabolites			
ROMt, gH_2O_2/100ml	5398 ± 21.83	2963 ± 13.09	1651 ± 5.35
AOPP, µmol/l	5161 ± 9.58	2929 ± 5.78	1646 ± 3.63
FRAP, µmol/l	2701 ± 26.17	1153 ± 11.56	464 ± 4.41
SHp, µmol/l	3103 ± 28.88	1432 ± 10.9	658 ± 4.53
Minerals			
Calcium, mmol/l	4164 ± 28.71	2075 ± 19.18	1053 ± 8.79
Phosphorus, mmol/l	3981 ± 20.13	1976 ± 9.11	991 ± 3.21
Magnesium, mmol/l	5908 ± 20.53	3287 ± 10.84	1790 ± 5.37
Potassium, mmol/l	4915 ± 17.80	2626 ± 8.30	1375 ± 4.89
Sodium, mmol/l	4481 ± 13.51	2318 ± 7.17	1172 ± 3.90
Chlorine, mmol/l	3795 ± 13.60	1824 ± 5.55	849 ± 2.93
Zinc, µmol/l	7202 ± 41.13	4223 ± 31.65	2490 ± 14.49

[1]NEFA - non-esterified fatty acids; BHB - β-hydroxybutyrate; AST - aspartate aminotransferase; GGT - γ -glutamyl transferase; BILt - total bilirubin; ALP - alkaline phosphatase; PON - paraoxonase; ROMt - total reactive oxygen metabolites; AOPP - advanced oxidation protein products; FRAP - ferric reducing antioxidant power; SHp – total thiol groups; PROTt - total proteins.

Table S3 – Average prediction performance (± SD) of milk AfiLab NIR alone (model 1, M1), considering the systematic effect of days in milk and parity (model 2, M2) and considering the systematic effect of days in milk, parity, and genomic information (model 3, M3) for the 5-fold random cross-validation scenario using the elastic net method for energy-related and liver function/hepatic damage blood metabolites.

Trait	AfiLab NIR (M1)						AfiLab NIR + Farm data (M2)						AfiLab NIR + Farm data + SNP marker (M3)					
	r	SD	RMSE	SD	Slope	SD	r	SD	RMSE	SD	Slope	SD	r	SD	RMSE	SD	Slope	SD
Energy-related metabolites																		
Glucose	0.47	0.060	0.27	0.020	0.88	0.150	0.51	0.032	0.28	0.020	0.94	0.090	0.66	0.026	0.22	0.050	0.99	0.070
Cholesterol	0.46	0.070	1.10	0.060	0.89	0.190	0.55	0.040	1.07	0.120	1.07	0.090	0.62	0.031	0.89	0.070	0.97	0.070
BHB	0.42	0.060	0.18	0.080	0.89	0.110	0.55	0.040	0.17	0.030	1.04	0.090	0.59	0.031	0.17	0.020	0.99	0.070
NEFA	0.31	0.070	0.15	0.100	1.15	0.150	0.41	0.020	0.09	0.010	0.99	0.110	0.45	0.040	0.13	0.050	0.95	0.070
Urea	0.54	0.060	0.60	0.040	1.01	0.090	0.58	0.040	0.85	0.060	0.96	0.080	0.63	0.018	0.79	0.060	1.01	0.030
Creatinine	0.40	0.070	5.50	1.370	1.06	0.060	0.46	0.030	4.95	0.940	0.96	0.120	0.50	0.070	4.89	0.910	0.96	0.110
Liver function and hepatic damage																		
Albumin	0.47	0.050	2.09	0.116	1.02	0.130	0.56	0.043	1.88	0.150	0.99	0.073	0.62	0.035	1.76	0.090	0.98	0.098
ALP	0.44	0.040	17.75	1.890	1.12	0.087	0.59	0.033	15.91	1.870	1.01	0.089	0.66	0.033	14.42	2.140	0.98	0.092
AST	0.43	0.045	21.19	4.623	0.94	0.085	0.47	0.073	20.73	2.490	0.97	0.085	0.52	0.035	20.52	6.280	0.99	0.061
GGT	0.57	0.040	6.89	1.170	1.01	0.086	0.61	0.060	6.64	1.213	1.02	0.081	0.64	0.022	6.33	1.090	0.97	0.067
BILt	0.37	0.057	1.08	0.287	0.91	0.102	0.44	0.053	1.00	0.300	0.97	0.092	0.49	0.033	0.98	0.130	1.00	0.083
PON	0.28	0.040	18.44	1.848	0.85	0.099	0.37	0.080	17.81	2.050	0.97	0.079	0.41	0.013	17.37	1.960	0.97	0.056

NEFA - non-esterified fatty acids; BHB - β-hydroxybutyrate; ALB – Albumin; ALP – alkaline phosphatase; BILt – total bilirubin; GGT – γ -glutamyl transferase; AST – aspartate aminotransferase; PON – paraoxonase

r – coefficient of correlation between the observed and predicted phenotypes in the validation set; SD – standard deviation; RMSE – root mean squared error; Slope - slope of the linear regression of predicted values ($\hat{y}$) on observed (y) value.

Table S4 – Average prediction performance (± SD) of milk AfiLab NIR alone (model 1, M1), considering the systematic effect of days in milk and parity (model 2, M2) and considering the systematic effect of days in milk, parity, and genomic information (model 3, M3) for the 5-fold random cross-validation scenario using the elastic net method for inflammation/innate immunity response and oxidative stress metabolites.

Trait	AfiLab NIR (M1)						AfiLab NIR + Farm data (M2)						AfiLab NIR + Farm data + SNP marker (M3)					
	r	SD	RMSE	SD	Slope	SD	r	SD	RMSE	SD	Slope	SD	r	SD	RMSE	SD	Slope	SD
Inflammation/innate immunity response																		
Ceruloplasmin	0.38	0.060	0.57	0.060	0.99	0.110	0.41	0.130	0.56	0.070	0.98	0.050	0.50	0.040	0.53	0.050	0.97	0.040
Haptoglobin	0.55	0.050	0.29	0.020	1.08	0.070	0.59	0.060	0.28	0.030	1.05	0.060	0.65	0.030	0.25	0.020	0.98	0.030
PROTt	0.55	0.060	3.93	0.360	0.99	0.110	0.58	0.170	3.92	0.280	0.97	0.090	0.62	0.050	3.73	0.370	0.96	0.060
Globulins	0.59	0.060	4.38	0.370	1.01	0.100	0.64	0.120	4.22	0.320	0.97	0.070	0.69	0.060	3.92	0.340	0.98	0.040
MPO	0.45	0.070	65.81	8.190	1.02	0.080	0.48	0.050	64.43	4.940	0.97	0.050	0.59	0.030	59.50	7.830	1.00	0.040
Oxidative stress metabolites																		
ROMt	0.34	0.044	8.57	1.731	1.00	0.095	0.54	0.047	2.72	0.280	1.01	0.082	0.61	0.047	2.40	0.290	0.98	0.056
AOPP	0.37	0.057	51.96	29.524	0.92	0.105	0.45	0.060	8.04	1.660	0.98	0.091	0.52	0.073	7.76	1.250	0.98	0.065
FRAP	0.52	0.041	2.73	0.364	0.90	0.042	0.54	0.047	47.72	18.290	0.95	0.034	0.61	0.047	41.01	12.280	0.97	0.033
SHp	0.48	0.053	45.59	3.277	1.01	0.107	0.54	0.033	43.06	3.150	0.99	0.075	0.63	0.054	39.34	3.940	0.96	0.048

MPO – myeloperoxidase; PROTt – total protein; ROMt – total reactive oxygen metabolites; AOPP – advanced oxidation protein products; FRAP – ferric reducing antioxidant power; SHp – total thiol groups.

r – coefficient of correlation between the observed and predicted phenotypes in the validation set; SD – standard deviation; RMSE – root mean squared error; Slope - slope of the linear regression of predicted values ($\hat{y}$) on observed (y) value.

Table S5 – Average prediction performance (± SD) of milk AfiLab NIR alone (model 1, M1), considering the systematic effect of days in milk and parity (model 2, M2) and considering the systematic effect of days in milk, parity, and genomic information (model 3, M3) for the 5-fold random cross-validation scenario using the elastic net method for blood minerals.

Trait	AfiLab NIR (M1)						AfiLab NIR + Farm data (M2)						AfiLab NIR + Farm data + SNP marker (M3)					
	r	SD	RMSE	SD	Slope	SD	r	SD	RMSE	SD	Slope	SD	r	SD	RMSE	SD	Slope	SD
Ca	0.26	0.045	0.11	0.013	0.98	0.099	0.41	0.040	0.10	0.010	0.95	0.075	0.47	0.057	0.10	0.010	0.96	0.060
P	0.27	0.027	0.32	0.021	0.95	0.061	0.43	0.033	0.30	0.020	1.00	0.060	0.50	0.060	0.29	0.020	1.05	0.033
Mg	0.41	0.045	0.09	0.012	1.00	0.085	0.44	0.060	0.09	0.010	0.98	0.080	0.50	0.033	0.09	0.010	0.99	0.037
K	0.39	0.046	0.33	0.042	0.87	0.075	0.40	0.067	0.33	0.040	0.99	0.065	0.49	0.040	0.32	0.020	1.00	0.043
Na	0.60	0.036	1.93	0.179	0.98	0.060	0.65	0.047	2.12	0.110	1.01	0.050	0.69	0.053	1.87	0.130	0.99	0.037
Cl	0.31	0.050	2.13	0.305	1.02	0.103	0.37	0.073	2.11	0.160	0.98	0.085	0.44	0.067	2.04	0.180	1.00	0.050
Zn	0.55	0.050	2.02	0.371	1.29	0.106	0.58	0.033	1.83	0.190	0.99	0.090	0.63	0.073	1.61	0.100	0.98	0.043

Ca – calcium; P – phosphorus; Mg– magnesium; K – potassium; Na – sodium; Cl – chlorine; Zn – zinc.
r – coefficient of correlation between the observed and predicted phenotypes in the validation set; SD – standard deviation; RMSE – root mean squared error; Slope - slope of the linear regression of predicted values ($\hat{y}$) on observed (y) value.
