# Supplementary material for: Combining genetic markers, on-farm information and infrared data for the in-line prediction of blood biomarkers of metabolic disorders in Holstein cattle
Source: J Anim Sci Biotechnol. 2024 Jun 9;15:83. doi: 10.1186/s40104-024-01042-3 (PMC11162571; doi:10.1186/s40104-024-01042-3)
Supplement: Supplementary file 2 — Additional file 2: Fig. S1. Relative difference (%) in predictive ability for 5-fold random cross-validation scenarios using Elastic-net for Model 2 (M2; milk NIR data and on-farm data) and Model 3 (M3; milk NIR data, on-farm and genomic information) against Model 1, which considers only the NIR infrared data. Data are shown as mean ± SD (red error bar line). Glu – glucose; Cholest – cholesterol; NEFA – non-esterified fatty acids; BHB – β-hydroxybutyrate; Crea – creatinine; AST – aspartate aminotransferase; GGT – γ-glutamyl transferase; BILt – total bilirubin; ALB – albumin; ALP – alkaline phosphatase; PON – paraoxonase; CuCp – ceruloplasmin; Glob – globulins; PROTt – total proteins; Hapto – haptoglobin; MPO – myeloperoxidase; ROMt – total reactive oxygen metabolites; AOPP – advanced oxidation protein products; FRAP – ferric reducing antioxidant power; SHp – total thiol groups; Ca – calcium; P – phosphorus; Mg – magnesium; K – potassium; Na – sodium; Cl – chlorine; Zn – zinc. Fig. S2. Relative gain in predictive ability Pearson correlation, considering three thresholds based on marker significance (-log10(P-value)) higher than 2.0, 2.5, and 3.0 against fitting all 61k SNPs, including standard errors, assessed for energy-related (a) and liver function and hepatic damage (b) blood metabolites. Data are shown as mean ± SD (black error bar line). Glu – glucose; Cholest – cholesterol; NEFA – non-esterified fatty acids; BHB – β-hydroxybutyrate; Crea – creatinine; AST – aspartate aminotransferase; GGT – γ-glutamyl transferase; BILt – total bilirubin; ALB – albumin; ALP – alkaline phosphatase; PON – paraoxonase. Fig. S3. Relative gain in predictive ability Pearson correlation, considering three thresholds based on marker significance (-log10(P-value)) higher than 2.0, 2.5 and 3.0 against fitting all 61k SNPs, including standard errors, assessed for inflammation/innate immunity response (a) and oxidative stress blood metabolites (b). Data are shown as mean ± SD (black error [file 40104_2024_1042_MOESM2_ESM.pdf]

Additional file 2

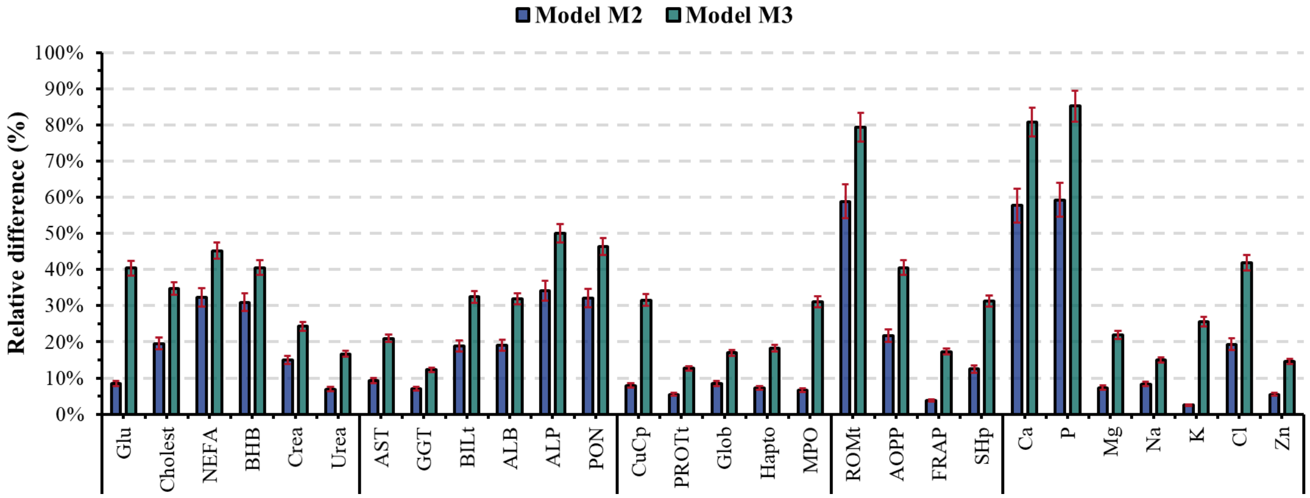

Fig. S1 – Relative difference (%) in predictive ability for 5-fold random cross-validation scenarios using Elastic-net for Model 2 (M2; milk NIR data and on-farm data) and Model 3 (M3; milk NIR data, on-farm and genomic information) against Model 1, which considers only the NIR infrared data. Data are shown as mean ± SD (red error bar line). Glu – glucose; Cholest – cholesterol; NEFA - non-esterified fatty acids; BHB - β-hydroxybutyrate; Crea – creatinine; AST – aspartate aminotransferase; GGT – γ -glutamyl transferase; BILt – total bilirubin; ALB – albumin; ALP – alkaline phosphatase; PON – paraoxonase; CuCp – ceruloplasmin; Glob – globulins; PROTt – total proteins; Hapto – haptoglobin; MPO – myeloperoxidase; ROMt – total reactive oxygen metabolites; AOPP – advanced oxidation protein products; FRAP – ferric reducing antioxidant power; SHp – total thiol groups; Ca – calcium; P – phosphorus; Mg– magnesium; K – potassium; Na – sodium; Cl – chlorine; Zn – zinc.

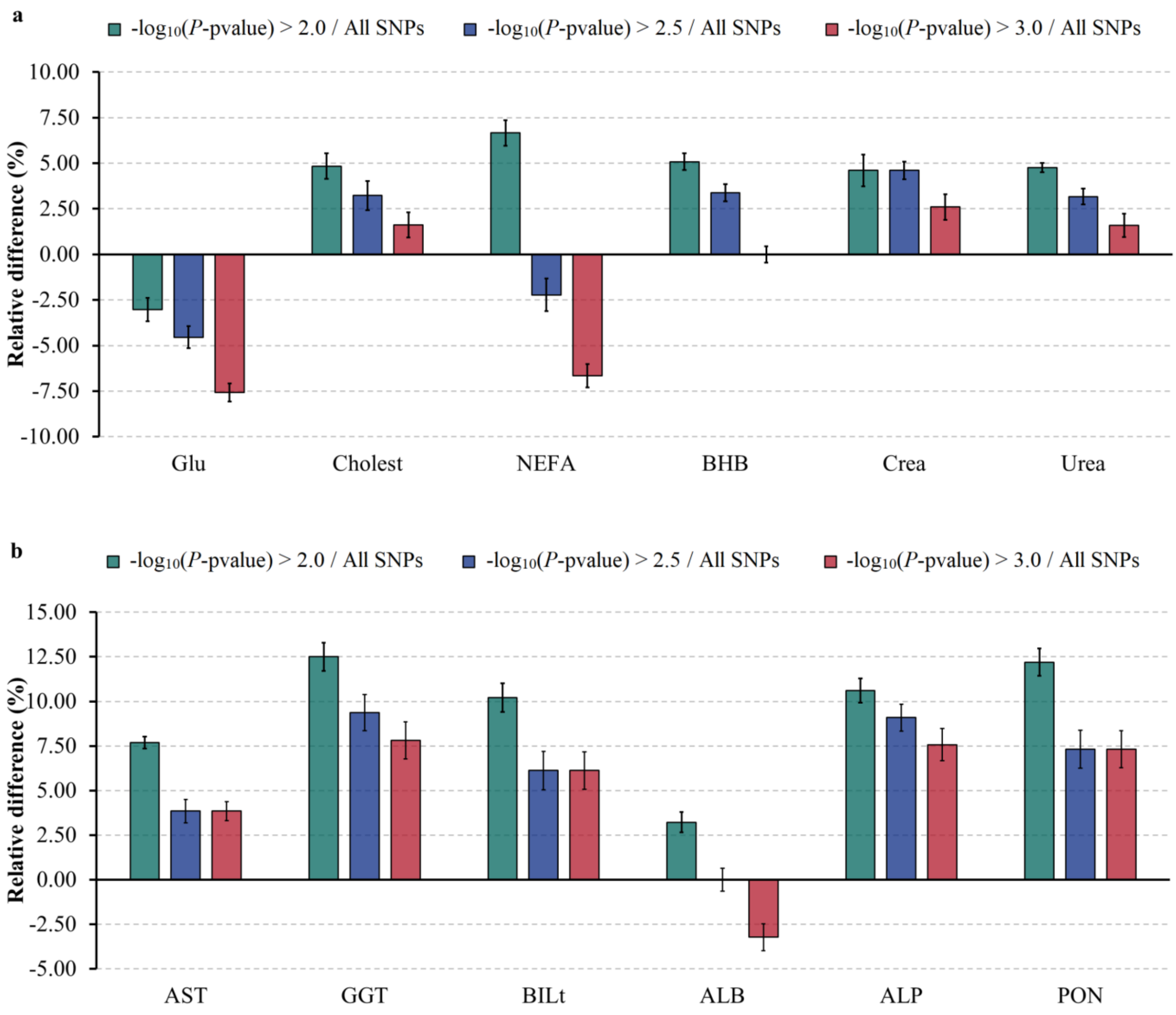

Fig. S2 – Relative gain in predictive ability Pearson correlation, considering three thresholds based on marker significance $(-\log_{10}(P - \text{value}))$ higher than 2.0, 2.5, and 3.0 against fitting all 61k SNPs, including standard errors, assessed for energy-related [A] and liver function and hepatic damage [B] blood metabolites. Data are shown as mean ± SD (black error bar line). Glu – glucose; Cholest – cholesterol; NEFA - non-esterified fatty acids; BHB - β-hydroxybutyrate; Crea – creatinine; AST – aspartate aminotransferase; GGT – γ -glutamyl transferase; BILt – total bilirubin; ALB – albumin; ALP – alkaline phosphatase; PON – paraoxonase.

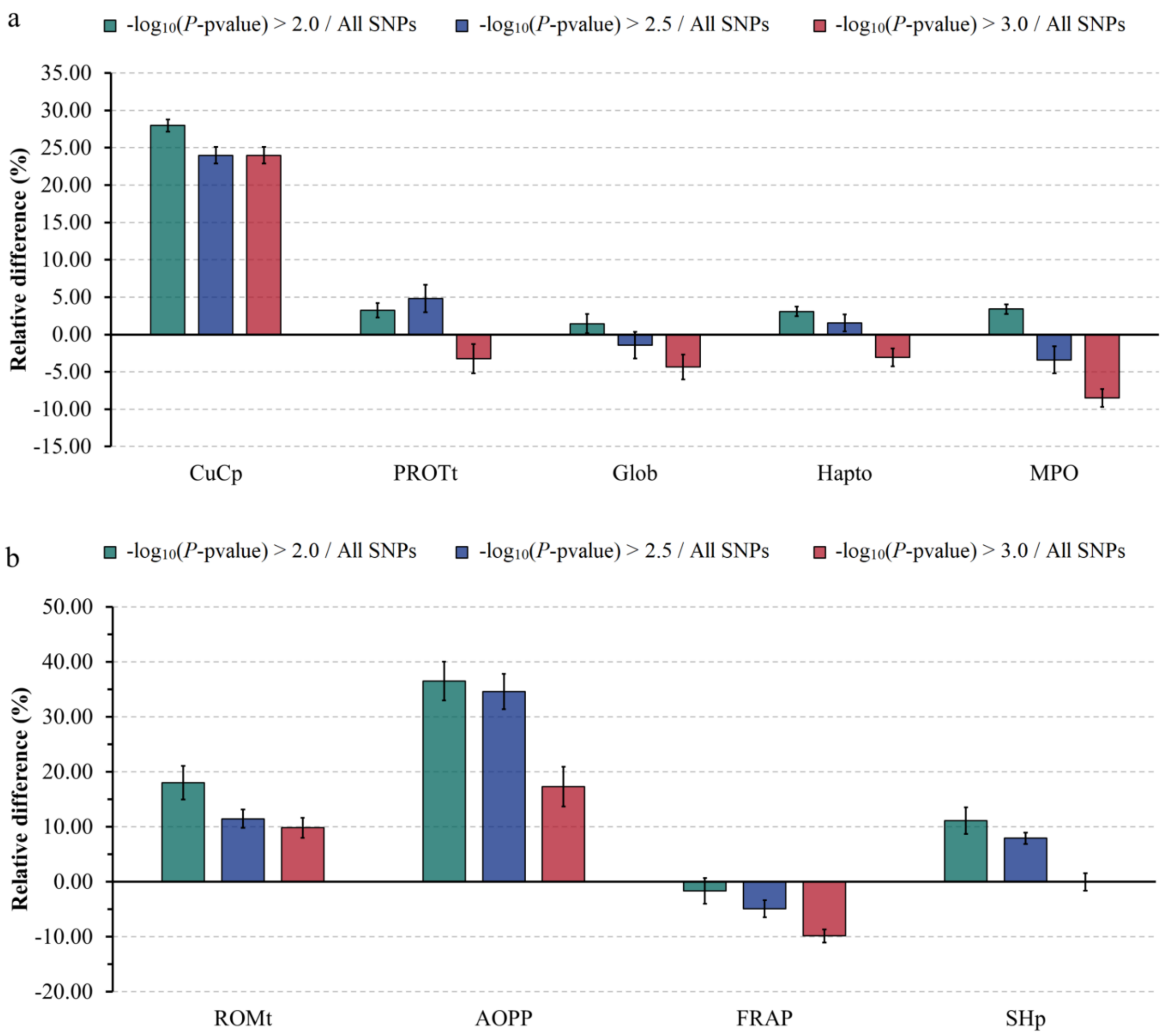

Fig. S3 – Relative gain in predictive ability Pearson correlation, considering three thresholds based on marker significance $(-\log_{10}(P - \text{value}))$ higher than 2, 2.5, and 3 against fitting all 61k SNPs, including standard errors, assessed for inflammation/innate immunity response [A] and oxidative stress blood metabolites [B]. Data are shown as mean ± SD (black error bar line). CuCp – ceruloplasmin; PROTt – total proteins; Glob – globulins; Hapto – haptoglobin; MPO – myeloperoxidase; ROMt – total reactive oxygen metabolites; AOPP – advanced oxidation protein products; FRAP – ferric reducing antioxidant power; SHp – total thiol groups.

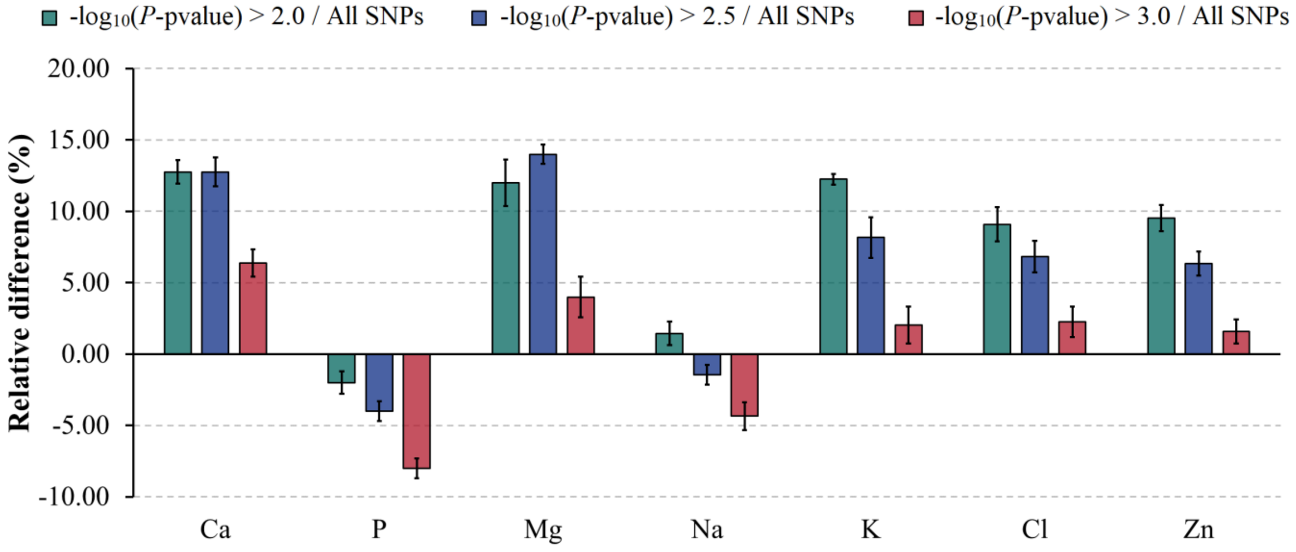

Fig. S4 – Relative gain in predictive ability Pearson correlation, considering three thresholds based on marker significance ($-\log_{10}(P - \text{value})$) higher than 2.0, 2.5, and 3.0 against fitting all 61k SNPs, including standard errors, assessed for blood minerals. Data are shown as mean ± SD (black error bar line). Ca – calcium; P – phosphorus; Mg – magnesium; Na – sodium; K – potassium; Cl – chlorine; Zn – zinc.

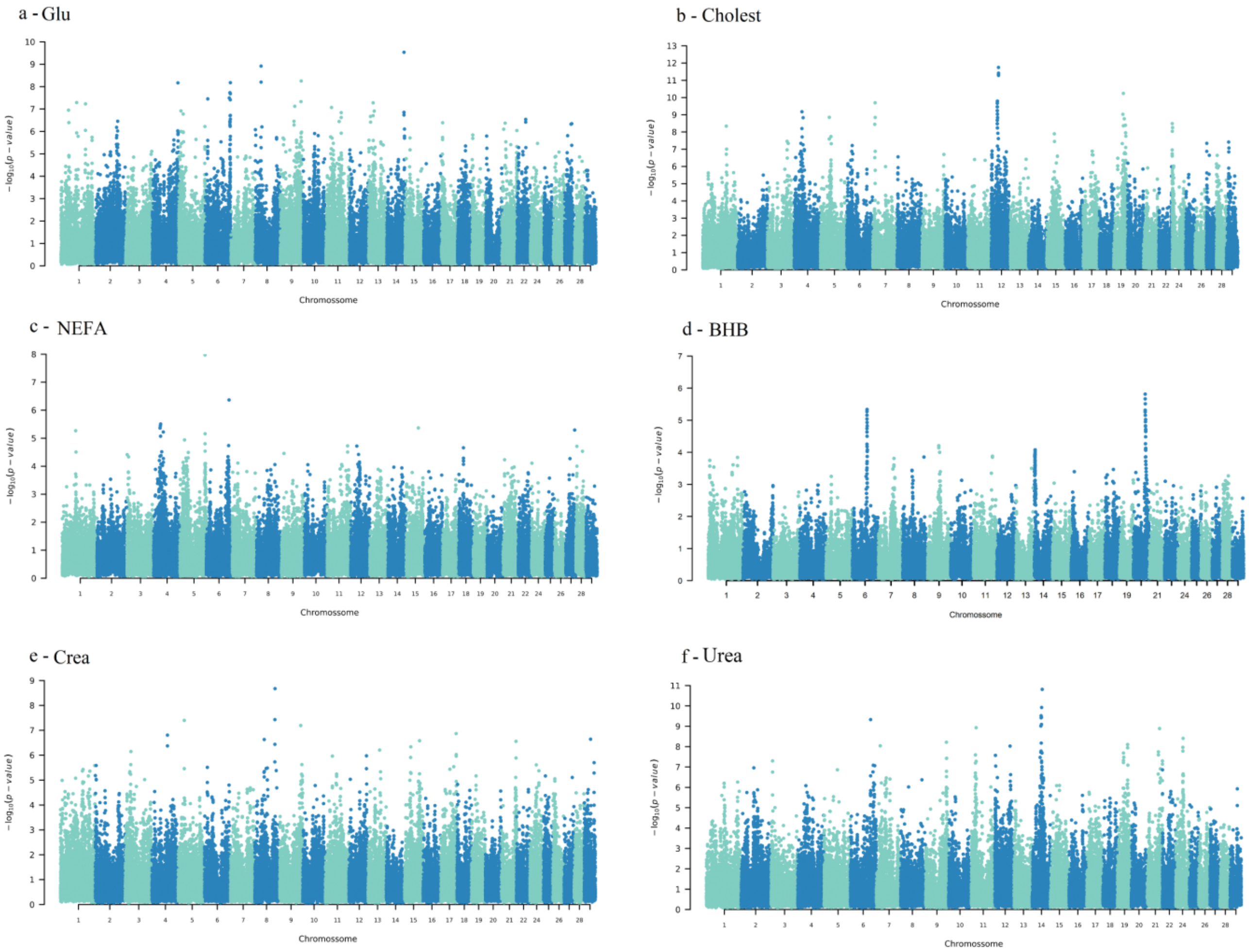

Fig. S5 – Manhattan plot for the average value of markers significance ($-\log_{10}(P - \text{value})$) across the 5-fold cross-validation for energy-related blood metabolites. Glu – glucose; Cholest – cholesterol; NEFA - non-esterified fatty acids; BHB - β-hydroxybutyrate; Crea – creatinine.

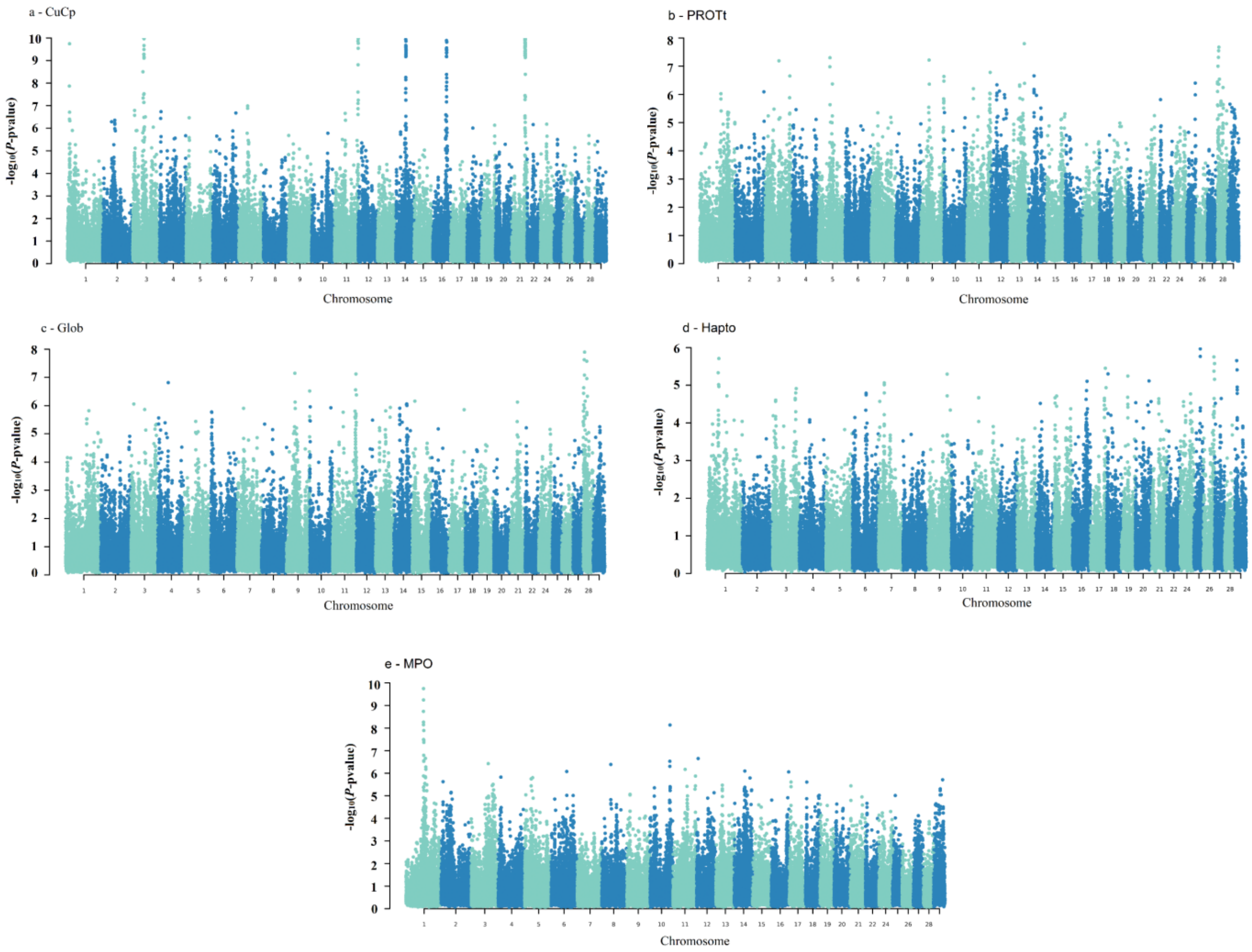

Fig. S6 - Manhattan plot for the average value of markers significance ($-\log_{10}(P - \text{value})$) across the 5-fold cross-validation for blood metabolites related to inflammation/innate immunity response. CuCp – ceruloplasmin; PROTt – total proteins; Glob – globulins; Hapto – haptoglobin; MPO – myeloperoxidase.

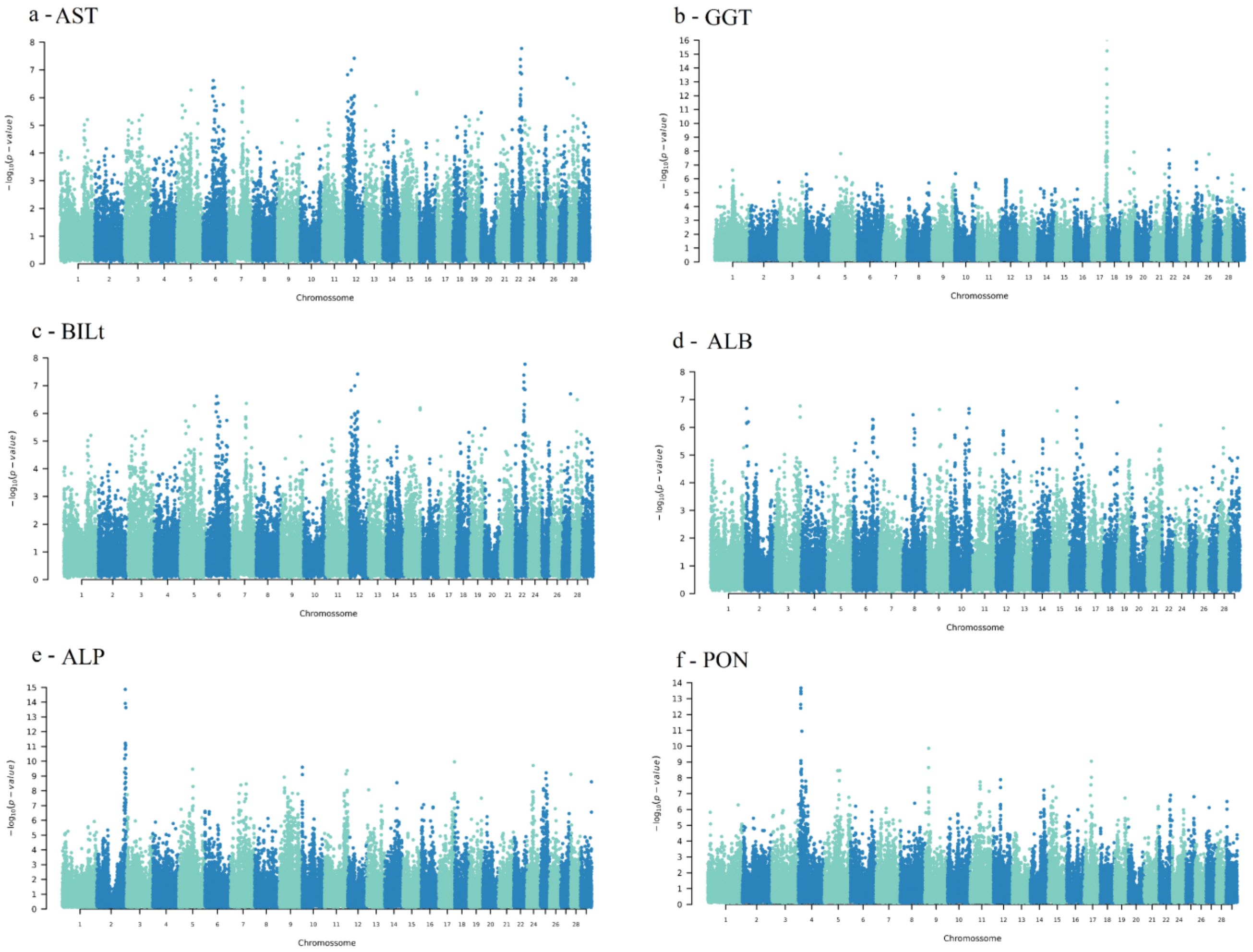

Fig. S7 – Manhattan plot for the average value of markers significance ($-\log_{10}(P - \text{value})$) across the 5-fold cross-validation for blood metabolites related to liver function and hepatic damage. AST – aspartate aminotransferase; GGT – γ -glutamyl transferase; BILt – total bilirubin; ALB – albumin; ALP – alkaline phosphatase; PON – paraoxonase.

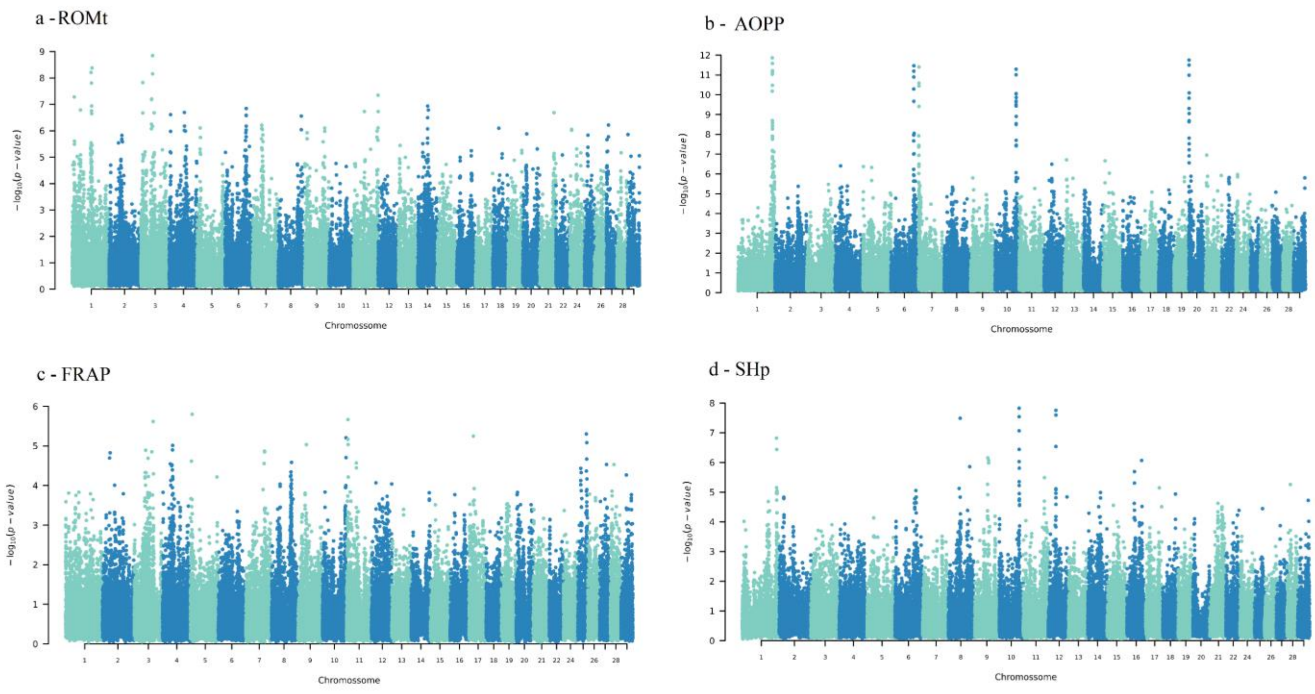

Fig. S8 - Manhattan plot for the average value of markers significance $(-\log_{10}(P - \text{value}))$ across the 5-fold cross-validation for oxidative stress blood metabolites. ROMt - total reactive oxygen metabolites; AOPP – advanced oxidation protein products; FRAP – ferric reducing antioxidant power; SHp – total thiol groups.

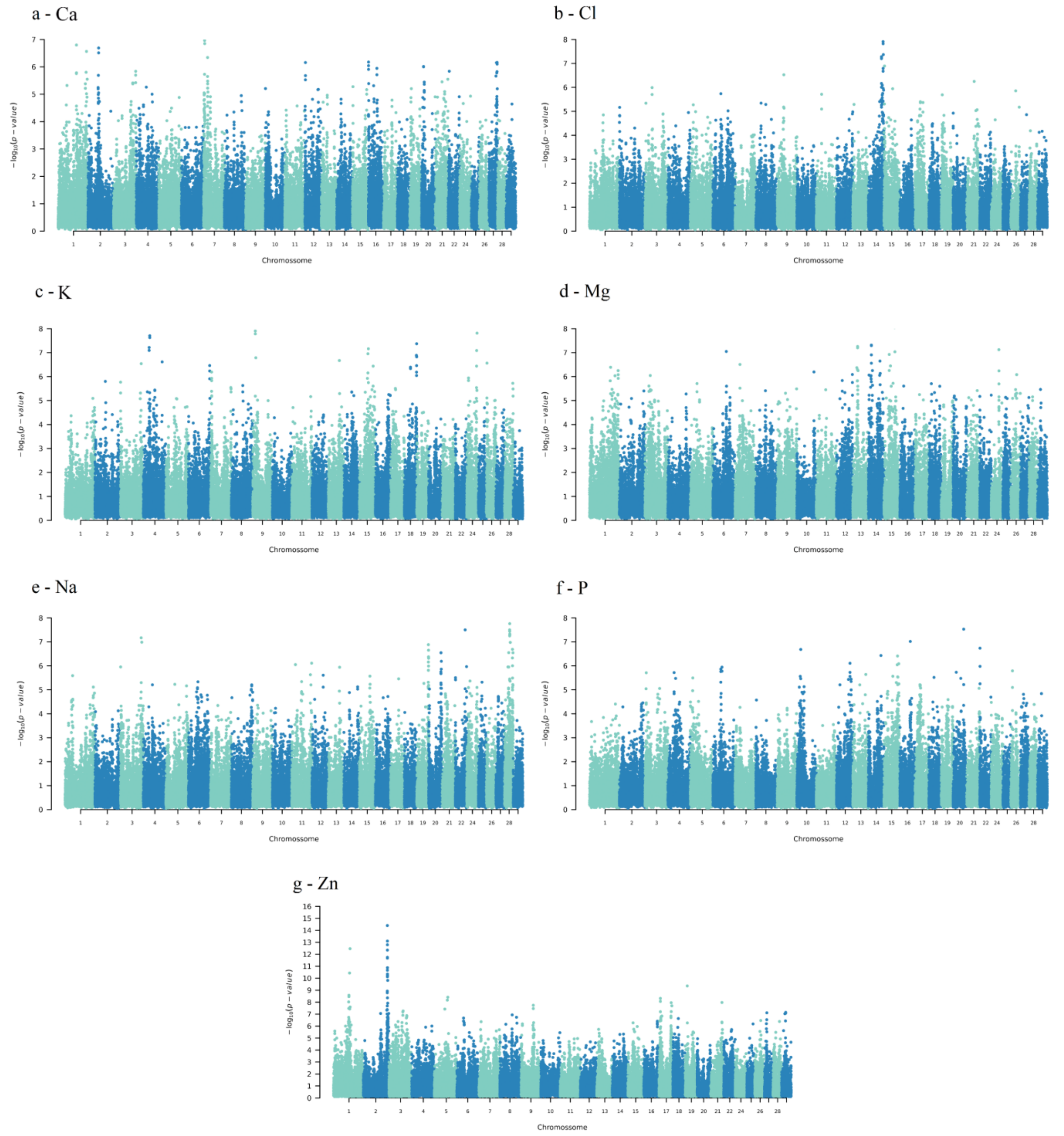

Fig. S9 - Manhattan plot for the average value of markers significance $(-\log_{10}(P - \text{value}))$ across the 5-fold cross-validation for blood minerals. Ca – calcium; P – phosphorus; Mg – magnesium; K – potassium; Na – sodium; Cl – chlorine; Zn – zinc.
